# Characterization of the genotypic and phenotypic spectrum of TCF7L2-related neurodevelopmental disorder (TRND)

**DOI:** 10.1016/j.gim.2026.102642

**Published:** 2026-07-07

**Authors:** Sally Nijim, Mimi Kim, Melissa Denish, Michael V. Gonzalez, Joseph Zinski, Claudine Rieubland, Dominique Braun, Elsebet Ostergaard, Amelle Shillington, Laurence Faivre, Julien Maraval, Aurore Garde, Christophe Philippe, Fréderic Tran-Mau-Them, Amy Crunk, UCI GREGoR Site, Megan Hawley, Bert Callewaert, Maria Iascone, Anna Cereda, Cecilia Daolio, Tova Hershkovitz, Jean-Marc Good, Katharina Steindl, Tanja Frey, Anita Rauch, Alexandra Afenjar, Cyril Mignot, Jean-Madeleine de Sainte Agathe, Nicolette den Hollander, Yvonne Hilhorst-Hofstee, Saskia Koene, Gijs Santen, Emilia K. Bijlsma, Sara Berger, Lakshmi Mehta, Radka Stoeva, Clara Houdayer, Paul Gueguen, Helene Faust, Sabine Specht, Annick Klabunde-Cherwon, Melik Malek Khelifa, Anke Bergmann, Carol Saunders, Magdalena Krygier, Diana Carrasco, Kay Metcalfe, Stephan J. Sanders, David Y. Zhang, Renae Judy, Wes Nijim, David Exposito-Alonso, Christine X. Deng, Jusung Kim, Jozef Gecz, Corrado Romano, Cindy Skinner, Angie Lichty, Ellen Linebaugh, Steven A. Skinner, Maria Chahrour, Tianyun Wang, Kun Xia, Hui Guo, Sien Van Daele, Gert Van Goethem, Christina Fagerberg, Jesper Graakjaer, Susanne Anders, Heike Fink, D. Isum Ward, Dorothy K. Grange, Alanna Strong, Petra Zwijnenburg, Meghan Towne, René G. Feichtinger, Jennifer Morrison, Aditi Dagli, Jonathan Levy, Yline Capri, Rebecca C. Spillman, Sarah Hart, Vandana Shashi, Boris Keren, Tjitske Kleefstra, Rolph Pfundt, Christian Gilissen, Evan E. Eichler, Melanie Brugger, Michael Zech, Wendy K. Chung, Maria Fasolino, Holly C. Dow, Daniel J. Rader, Edward S. Brodkin, Maja Bucan, Eric D. Marsh, Caroline Dias, David C. Fajgenbaum

**Affiliations:** 1. Division of Translational Medicine & Human Genetics & Center for Cytokine Storm Treatment & Laboratory, Perelman School of Medicine, University of Pennsylvania, 3535 Market Street, Suite 700, Philadelphia, PA 19104; 2. Sidney Kimmel Medical College, Thomas Jefferson University, 1025 Walnut St, Suite 100, Philadelphia, PA 19107; 3. Department of Medical Genetics, Central Institute of the Hospitals, Hospital of the Valais, Avenue Grand-Champsec 86,1951 Sion, Switzerland; 4. Department of Human Genetics, Inselspital Bern, University of Bern, 3010 Bern, Switzerland; 5. Copenhagen University Hospital Rigshospitalet Blegdamsvej, Department of Genetics, 2100 Copenhagen, Denmark; 6. Department of Clinical Medicine, University of Copenhagen, Copenhagen, Denmark; 7. Cincinnati Children’s Hospital Medical Center, Division of Human Genetics, 3333 Burnet Avenue, Cincinnati, Ohio; 8. Université Bourgogne Europe, CHU Dijon Bourgogne, Inserm, CTM UMR1231, équipe GAD, FHU TRANSLAD, Centre de génétique, Centre de référence Anomalies du Développement et Syndromes Malformatifs, 21000 Dijon, France; 9. Université Bourgogne Europe, CHU Dijon Bourgogne, Inserm, CTM UMR1231, équipe GAD, FHU TRANSLAD, Centre de référence GénoPsy, 21000 Dijon, France.; 10. Université Bourgogne Europe, CHU Dijon Bourgogne, Inserm, CTM UMR1231, équipe GAD, FHU TRANSLAD, Centre de génétique, Centre de référence Déficiences Intellectuelles de Causes Rares, 21000 Dijon, France.; 11. Université Bourgogne Europe, CHU Dijon Bourgogne, Inserm, CTM UMR1231, équipe GAD, FHU TRANSLAD, Service de Génomique Médicale, 21000 Dijon, France.; 12. GeneDx, LLC, Gaithersburg, MD, 20877 USA; 13. Labcorp Genetics Inc, Burlington, North Carolina, USA; 14. Ghent University Hospital, Center for Medical Genetics, De Pintelaan 185, 1st Floor, Room 110.029, 9000 Ghent, Belgium; 15. Laboratorio di Genetica Medica, ASST Papa Giovanni XXIII, 24127 Bergamo, Italy; 16. Department of Pediatrics Papa Giovanni XXIII Hospital, Bergamo, Italy; 17. Institute of Human Genetics, Galilee Medical Center, 89 Nahariya-Cabri, Nahariya, Israel; 18. Division of Genetic Medicine, Lausanne University Hospital (CHUV) and University of Lausanne, av. Pierre Decker 5, CH-1011 Lausanne, Switzerland; 19. Universität Zürich, Institut für Medizinische Genetik, Wagistrasse 12, CH-8953 Schlieren, Switzerland; 20. APHP.Sorbonne Université, Département de Génétique, GH Pitié-Salpêtrière et Hôpital Trousseau, & Centre de Référence Déficiences Intellectuelles de Causes Rares, Paris, France; 21. Laboratoire de Biologie Médicale Seqoia, Paris, France; 22. Leiden University Medical Center, Postbus 9600, 2300 RC Leiden, The Netherlands; 23. Division of Clinical Genetics, Department of Pediatrics, Columbia University Vagelos College of Physicians and Surgeons, 3959 Broadway CHN7-718, New York, NY, USA; 24. Le Mans Hospital, Department of Medical Genetics, 194, avenue Rubillard, 72037, Le Mans, France; 25. Service de Génétique Médicale, CHU d’Angers, Angers, France; 26. Service de Génétique, CHRU de Tours, Tours, France; 27. Université de Tours, INSERM, Imaging Brain & Neuropsychiatry iBraiN U1253, Tours, France; 28. Institute of Human Genetics, University Hospital Leipzig, Phillip-Rosenthal-Straße 55, 04103 Leipzig, Germany; 29. Institute of Human Genetics, University Hospital Schleswig-Holstein, Arnold-Heller-Straße 3, 24105 Kiel, Germany; 30. Heidelberg University, Medical Faculty Heidelberg, Center for Pediatric and Adolescent Medicine, Department I, Division of Pediatric Neurology and Metabolic Medicine; 31. Heidelberg University, Medical Faculty Heidelberg, Center for Pediatric and Adolescent Medicine, Department I, Division of Pediatric Epileptology; 32. Department of Human Genetics, Hannover Medical School, Carl-Neuberg-Straße 1, 30625 Hannover, Germany; 33. Institute of Human Genetics, Hannover Medical School, Carl-Neuberg-Straße 1, 30625 Hannover, Germany; 34. University Hospital Würzburg, Clinical Genetics and Genomic Medicine, Josef-Schneider-Str. 2, 97080, Würzburg, Germany; 35. Department of Pathology and Laboratory Medicine, Children’s Mercy Hospital, Kansas City, MO, United States, 64108; 36. University of Missouri Kansas City, School of Medicine, Kansas City, MO, United States, 64108; 37. Department of Developmental Neurology, Medical University of Gdańsk, Dębinki 7, 80-952 Gdańsk, Poland; 38. Cook Children’s Medical Center, Clinical Genetics, 750 8th Ave #200, Fort Worth, Texas 76104; 39. Manchester Centre for Genomic Medicine, Manchester University NHS Foundation Trust, Manchester, United Kingdom; 40. Institute of Developmental and Regenerative Medicine, Department of Paediatrics, University of Oxford, Oxford, OX3 7TY, UK; 41. Department of Psychiatry and Behavioral Sciences, UCSF Weill Institute for Neurosciences, University of California, San Francisco, San Francisco, CA 94158, USA; 42. University of Pennsylvania, 3451 Walnut St, Philadelphia, PA 19104; 43. The University of Illinois at Chicago, Chicago, Illinois; 44. Division of Genetics and Genomics, Department of Pediatrics, Boston Children’s Hospital, Harvard Medical School, Boston, MA, USA; 45. Howard Hughes Medical Institute, Boston Children’s Hospital, Harvard Medical School, Boston, MA, USA; 46. Drexel University School of Medicine, 60 N. 36th Street, Philadelphia, PA 19104; 47. Adelaide Medical School, The University of Adelaide, 4 North Terrace, Adelaide SA 5000, Australia; 48. University of Catania, Department of Biomedical and Biotechnological Sciences, Section of Clinical Biochemistry and Medical Genetics, Via Santa Sofia, 97, 95123 Catania CT, Italy; 49. Oasi Research Institute-IRCCS, Research Unit of Rare Diseases and Neurodevelopmental Disorders, Via Conte Ruggero, 73, 94018 Troina EN, Italy; 50. JC Self Research Institute, 113 Gregor Mendel Circle, Greenwood, SC 29646; 51. University of Texas Southwestern Medical Center, 5323 Harry Hines Blvd, Dallas, TX 75390; 52. Department of Medical Genetics, Center for Medical Genetics, School of Basic Medical Sciences, Peking University, Beijing 100191, China.; 53. Neuroscience Research Institute, Peking University; Key Laboratory for Neuroscience, Ministry of Education of China & National Health Commission of China, Beijing 100191, China.; 54. Autism Research Center, Peking University Health Science Center, Beijing 100191, China.; 55. Hunan Key Laboratory of Medical Genetics & MOE Key Laboratory of Rare Pediatric Diseases, School of Life Sciences, Central South University, Changsha, Hunan 410078, China; 56. Department of Human Genetics, University Hospitals Leuven, 3000 Leuven, Belgium; 57. University of Antwerp, Department of Neurology, Prinsstraat 13, 2000 Antwerpen, Belgium; 58. Hospital of Lillebaelt, Vejle Hospital, Clinical Genetics, 7100 Vejle, Denmark; 59. MVZ Mitteldeutscher Praxisverbund Humangenetik, Friedrichstraße 38-40, 01067 Dresden, Germany; 60. Sanford Children’s Specialty Clinic, 1600 W. 22nd St., Sioux Falls, South Dakota 57105; 61. Washington University School of Medicine, 660 S. Euclid Ave., St. Louis, MO 63110; 62. Division of Human Genetics, Children’s Hospital of Philadelphia, Philadelphia, Pennsylvania, USA; 63. Center for Applied Genomics, Children’s Hospital of Philadelphia, Philadelphia, Pennsylvania, USA; 64. Department of Pediatrics, Perelman School of Medicine at the University of Pennsylvania, Philadelphia, Pennsylvania, USA; 65. Amsterdam University Medical Center, Meibergdreef 9, 1105 AZ Amsterdam; 66. Ambry Genetics Corporation, Aliso Viejo, CA; 67. University Children’s Hospital, Salzburger Landesklinken (SALK) and Paracelsus Medical University (PMU), Müllner Hauptstraße 48, 5020 Salzburg, Austria; 68. Arnold Palmer Hospital for Children, 60 W. Gore St 2^nd^ floor, MP 152, Orlando, FL 32806; 69. Hôpital Universitaire Robert-Debré, AP-HP. Nord – Université de Paris, 48 Boulevard Serurier 75019 Paris; 70. Duke University Medical Center, 200 Trent Drive, Durham, NC 27710; 71. AP-HP.Sorbonne Université, Hôpital de la Pitié-Salpêtrière, 47-83 boulevard de l’Hôpital, 75013, Paris, France; 72. Department of Genome Sciences, University of Washington School of Medicine, Seattle, WA 98195, USA; 73. Howard Hughes Medical Institute, University of Washington, Seattle, WA 98195, USA; 74. School of Medicine, Institute of Human Genetics, Klinikum rechts der Isar, Technical University of Munich, Munich, Germany; 75. Institute of Neurogenomics, Helmholtz Munich, Munich, Germany; 76. Institute for Advanced Study, Technical University of Munich, Munich, Germany; 77. Boston Children’s Hospital, Harvard Medical School, 300 Longwood Avenue, Boston, MA 02115; 78. University of Pennsylvania Autism Spectrum Program of Excellence, 125 South 31st Street, Philadelphia, PA 19104; 79. Children’s Hospital of Philadelphia, 3401 Civic Center Boulevard, Philadelphia, PA 19104; 80. University of Colorado Anschutz Medical Campus, Aurora, CO 80045

**Keywords:** TCF7L2, TCF7L2-related neurodevelopmental disorder (TRND), neurodevelopmental syndrome, speech delay

## Abstract

**Purpose:**

*TCF7L2* (OMIM:602228; HGNC:11641) is a transcription factor and critical effector of the *Wnt/β*-Catenin pathway. In 2021, 11 pediatric patients with mono-allelic predicted loss-of-function (pLOF) *TCF7L2* variants and syndromic features were observed. Characterization of patients with pLOF *TCF7L2* variants and neurodevelopmental features – herein referred to as *TCF7L2*-related neurodevelopmental disorder (TRND) – is urgently needed.

**Methods:**

We leveraged multiple methods (GeneMatcher, DECIPHER, literature review, public/private repositories) to identify an international cohort of 76 patients with pLOF *TCF7L2* variants and neurodevelopmental features and phenotypically characterized them. We also retrospectively searched for an independent cohort of adults with pLOF *TCF7L2* variants (n = 11) from 60,000+ PennMedicine BioBank (PMBB) patients.

**Results:**

Among 76 patients with pLOF *TCF7L2* variants, speech delay (95.3%), craniofacial dysmorphisms (73.3%), ophthalmologic conditions (65.5%), autism (62.1%), and orthopedic abnormalities (52.6%) were most commonly observed. Phenotypic differences did not cluster by variant type or genomic locus. Among PMBB patients, an association of nominal significance with type 2 diabetes with renal manifestations (OR = 5.8; p-value = 0.03) was detected, warranting further investigation.

**Conclusions:**

This represents the most comprehensive characterization to date of TRND, a novel neurodevelopmental disorder, defining its genotypic and phenotypic spectrum. We opened a Simons Searchlight natural history study (https://www.simonssearchlight.org/research/what-we-study/tcf7l2/) to enhance understanding of this condition.

## Introduction

Transcription factor 7-like 2 (*TCF7L2*) (OMIM:602228; HGNC:11641) – formerly TCF4-transcription factor 4 – is a key mediator of canonical *Wnt/β*-catenin signaling across human biology and disease^1,2^. In fact, genome-wide association studies have linked *TCF7L2* single nucleotide variants (SNVs) with increased risk for type 2 diabetes mellitus (T2D), colon cancer, bipolar disorder, schizophrenia, and autism spectrum disorder (ASD)^2–4^. *TCF7L2* is located on chromosome 10q25.2, and the encoded protein is a member of the TCF/LEF (T-cell factor/lymphoid enhancer binding factor) family of proteins, which complex with beta-catenin (CTNNB1) to bind DNA via a highly conserved high-mobility group (HMG Box) domain^1,5^. Transcriptional regulation of *Wnt*-responsive genes is vital for fetal development and adult tissue homeostasis^1–2^. *TCF7L2*, specifically, has a complex splicing pattern that generates distinct protein isoforms – each with agonistic and antagonistic activities on transcriptional regulation of canonical Wnt/*β*-catenin signaling pathways^2,6^.

*TCF7L2* is especially critical for central nervous system (CNS) development. In fact, *TCF7L2* homozygous knockout in mice results in neural tube duplications and severe truncations of caudal endoderm incompatible with life^7^. Single-cell RNA sequencing of human neuronal tissue has demonstrated *TCF7L2* expression in several CNS cell populations throughout development^8^, and *TCF7L2* has been implicated in astrocyte maturation^8–9^, thalamic and midbrain development^10–11^, and premyelination and maturation of oligodendrocytes^12–13^. In humans, *TCF7L2* has high intolerance to loss-of-function variation (probability of loss-of-function intolerance (pLI) = 1). Several large-scale studies over the past decade have identified occasional patients with intellectual disability (ID) and autism spectrum disorder (ASD) with variants in *TCF7L2*^14–19^.

Recently, Dias et al.^20^ reported a small case series of 11 pediatric patients with *de novo* heterozygous nonsense, splice-site, and missense variants in *TCF7L2* with neurodevelopmental syndromic features. This small cohort all had developmental delays in addition to a range of ophthalmologic, orthopedic, and neuropsychiatric comorbidities, such as ASD and attention-deficit/hyperactivity disorder (ADHD). All variants were in highly conserved regions expected to disrupt *TCF7L2* function. Functional mouse models with two of these variants demonstrated reduced *TCF7L2* HMG Box binding, anxiety-like phenotypes, and impaired vocalization consistent with previous murine ASD models^21^. These findings collectively suggest *TCF7L2* haploinsufficiency contributes to risk for a novel neurodevelopmental disorder, herein referred to as *TCF7L2*-related neurodevelopmental disorder (TRND).

Given the small sample size and limited research to date, many questions remain, including those regarding TRND’s natural history and phenotypic and genotypic spectrum. In this study, we sought to comprehensively establish this for future investigation.

## Methods

During this retrospective cohort study, we identified patients with predicted loss-of-function (pLOF) variants in *TCF7L2* from 06/2022–10/2024 (Figure 1). We also opened a Simons Searchlight natural history study (https://www.simonssearchlight.org/research/what-we-study/tcf7l2/) currently available for prospective patient enrollment.

### Identifying and classifying patients with predicted loss-of-function TCF7L2 variants

Orthogonal identification strategies were implemented, including: (i) patient identification with GeneMatcher^22^ through the Matchmaker Exchange Network, (ii) initiating contact with clinicians of cases published in DECIPHER^23^, (iii) collating patient data from public/private repositories (Simons Simplex Collection (SSC)^24^, Simons Foundation Autism Research Initiative (SFARI)^24^, ClinVar^25^, Ambry Genetics), and (iv) outreach to authors/clinicians of cases yielded from systematic literature reviews. Literature review was conducted using the keyword search term “TCF7L2” on PubMed, and corresponding authors were contacted regarding potential cases. Following initial identification (n = 125), 37 cases were excluded as they were either (i) identified as duplicates under different source-specific identifiers and removed to ensure patients were uniquely represented or (ii) outside of the coding sequence (e.g., intron variants).

Patients with *TCF7L2* variants were classified as pLOF and “Likely TRND” (Supplemental Table 1) if: (a) any neurodevelopmental phenotype was reported, (b) the patient had no other variants known to contribute to a neurodevelopmental phenotype (per report using patient-specific identification methods listed in Supplemental Table 1), and (c) the following inclusion criteria for each variant class were met: (i) nonsense variants were located ≥50 nucleotides upstream of the final exon-exon junction, and, thus, expected to cause nonsense-mediated decay of the transcript^20,28^; (ii) splice-site variants scoring highly on splicing prediction tools (MaxEntScan^26^ |delta score|≥8 or SpliceAI^27^ score≥0.8); (iii) out-of-frame indel variants expected to introduce a stop codon ≥50 nucleotides upstream of the final exon-exon junction^20,28^; (iv) missense variants with <0.01% total allele frequency in gnomADv4.1.0^29^ and with scores supportive of pathogenicity in at least 2/4 predictive tools (AlphaMissense^30^ 0.56–1.0, ClinPred^31^≥0.5, PolyPhen^32^ “possibly” or “probably damaging”≥0.446, and/or PHRED-like CADD^33^≥25); (v) CNV deletion variants expected to disrupt *TCF7L2* not otherwise spanning genes known to explain the presenting phenotype; or (vi) in-frame indel variants not present in gnomAD^29^ in a highly conserved region. Pathogenicity scores generated using prediction algorithms for missense and splice-site variants are summarized in Supplemental Table 2. Some identified patients had suspicious *TCF7L2* variants and no other clear genetic contributor for their neurodevelopmental phenotype but did not meet aforementioned criteria. These cases were separately classified as “Suspected TRND,” with case-specific reasons for exclusion in Supplemental Table 4.

Genetic variants were primarily identified using exome sequencing and/or Sanger sequencing (n = 49). Remaining cases were identified using non-Sanger genome sequencing (n = 7), other non-Sanger assays (n = 6; molecular inversion probe assay, expanded gene panel, microarray, and/or FISH), or were otherwise unable to be confirmed due to case-specific limitations (n = 14). All reported variants were converted to the MANE select transcript (NM_001367943.1) using the Ensembl Variant Effect Predictor tool^34^. An overview of patient identification and classification is summarized in Figure 1. Composite genotypic and phenotypic information for “Likely TRND” and “Suspected TRND” cases is available in Supplemental Tables 1 and 4, respectively.

### Phenotypic characterization of TRND patients

Following patient identification, clinicians were provided a tabular survey (Supplemental Table 3) to complete with de-identifiable data regarding their case, including free-text response questions regarding genotypic, phenotypic, and historical details. Of the 76 “Likely TRND” and 12 “Suspected TRND” patients, 20 and 7, respectively, could not be further characterized through the tabular survey by the referring clinician/site/author, and only publicly available data were used in these cases. Written responses were clarified during follow-up and phenotypic information was standardized using the Human Phenotype Ontology (HPO)^35^ during analysis. Translated phenotypic details were corroborated with clinicians and updated at the study’s conclusion. To characterize the spectrum of features shared across “Likely TRND” patients, we extracted HPO-standardized terms that co-occurred in at least 2 patient cases, comprising 81 features (Figure 1, Supplemental Table 1). 10 broad categories of non-neurodevelopmental phenotypic features were additionally included in analysis (Supplemental Table 1).

### Interrogation of adult biobank and EHR data for individuals with pLOF TCF7L2 variants

We also conducted a retrospective screen of the PennMedicine Biobank (PMBB) at the University of Pennsylvania – a repository linked to the PennMedicine electronic health record (EHR) system – to identify adults with predicted *TCF7L2* loss-of-function variants. The biobank includes approximately 60,000 adult patient volunteers, who have had a genome-wide SNP array performed, a subset of whom also received exome sequencing. Predicted loss of function (pLOF) variants in *TCF7L2* were defined as frameshift, splicing, or stop-gain variants, as annotated by ANNOVAR^36^, and filtered for pathogenicity using REVEL^37^ scores ≥0.5. Following enforcement of this criterion, 12 different heterozygous pLOF variants in *TCF7L2* were detected among an initial pool of 22 PMBB patients. 11 patients had nonsense or out-of-frame indel variants not meeting the ≥50 nucleotides before the last exon-exon junction threshold criterion and were excluded, resulting in a final n = 11 sample size. Age/sex-matched controls were identified by selecting patients with common variants in *TCF7L2* (allele frequency ≥10%). PCA-based matching on Euclidean distance was used to randomly select approximately 10 controls/case from the n = 22 unfiltered dataset (n = 198 controls). All diagnoses in the EHR for each patient were recorded, as well as any ICD-9 codes. Median age and BMI were reported relative to date of last encounter. PMBB “phecodes”, groups of ICD-9 codes depicting clinically meaningful phenotypes, were compared between control and pLOF cohorts. Statistical significance was evaluated using Fisher’s exact tests with Benjamini-Hochberg correction for categorical variables and Mann-Whitney U tests for quantitative variables.

## Results

### pLOF TCF7L2 variants in highly conserved protein domains are present in TRND

76 patients were identified as “Likely TRND,” with nonsense (n = 10), out-of-frame indel (n = 18), missense (n = 33), splice-site (n = 10), in-frame indel (n = 1), and CNV (n = 4) variants in *TCF7L2*. The median patient age at the most recent recorded clinical assessment was 8.17 years (interquartile range (IQR) = 3.94–13.25) and 55.71% were male. Most patients for which reported ethnicity was available were identified by clinicians as White Non-Hispanic (39.47%). An additional 12 patients were identified as “Suspected TRND,” with insufficient evidence to meet criteria. Features of both cohorts are included in Table 1.

Most variants (n=72) were predicted to impact the coding sequence of (1) the full-length isoforms of *TCF7L2* (FL-*TCF7L2*) and (2) the short isoforms of *TCF7L2* lacking the N-terminal β-catenin binding domain that act as a dominant-negative antagonist of canonical Wnt signaling (DN-*TCF7L2*). However, 4 cases (P11, P17, P18, P21) included variants prior to exon 4, predicted to impact only FL-*TCF7L2* and lncRNA, excluding DN-*TCF7L2* isoforms (NM_001349870.2, NM_001349871.1).

Of the 76 identified variants, 7 were shared by 2 patients each (14 patients total). The 7 shared variants among “Likely TRND” cases were: (1) NM_001367943.1:c.1268A>G, NP_001354872.1:p.(Tyr423Cys), NG_012631.1:g.207190A>G, NC_000010.11:g.113152439A>G; (2) NM_001367943.1:c.1001+1G>A, NP_001354872.1:p.?, NG_012631.1:g.205875G>A, NC_000010.11:g.113151124G>A; (3) NM_001367943.1:c.634_635dup, NP_001354872.1:p.(Pro213ArgfsTer13), NG_012631.1:g.196016_196017dup, NC_000010.11:g.113141265_113141266dup; (4) NM_001367943.1:c.162del, NP_001354872.1:p.(Asn55IlefsTer53), NG_012631.1:g.5669del, NC_000010.11:g.112950918del; (5) NM_001367943.1:c.686–1G>A, NP_001354872.1:p.?, NG_012631.1:g.198673G>A, NC_000010.11:g.113143922G>A; (6) NM_001367943.1:c.1172T>C, NP_001354872.1:p.(Leu391Pro), NG_012631.1:g.207094T>C, NC_000010.11:g.113152343T>C; and (7) NM_001367943.1:c.1604C>T, NP_001354872.1:p.(Ser535Leu), NG_012631.1:g.220467C>T, NC_000010.11:g.113165716C>T. All other variants were found in unique patients. The spectrum of *TCF7L2* variants were mapped to the MANE select transcript, NM_001367943.1 (Figure 2). Patient cases with “Likely TRND” clustered at the C-terminal ends of the CTNNB1 binding domains and HMG Box domain. Variants were also clustered at a highly conserved region between both domains.

### TRND patients most frequently presented with developmental, ophthalmologic, and orthopedic abnormalities

Of the 76 “Likely TRND” patients, they were marginally heavier and relatively average height compared to reference populations – with median weight and height Z-scores of 0.85 (IQR = −0.25–1.60) and 0.14 (−0.95–0.66), respectively. 81 HPO-standardized ontology terms were described by clinicians in at least 2 TRND patients as well as 10 broader categories of non-HPO, non-neurodevelopmental features. HPO codes associated with each term are listed in Supplemental Table 1. Among “Likely TRND” cases (Figure 3A–B, Supplemental Figure 1), nearly all presented with speech delay (61/64, 95.31%). Other frequently reported neurodevelopmental phenotypes included ASD (36/58, 62.07%, OMIM:209850), gross motor delay (29/58, 50.0%), ID (27/56, 48.21%), ADHD/ADD (20/47, 42.55%, OMIM:143465), learning disability (9/32, 28.13%), and fine motor delay (12/54, 22.22%). When degree of ID was clinician-reported or IQ scores were provided, most patients with ID were in the mild-to-moderate range (11/16, 68.75%); a minority (5/16, 31.25%) were classified as “severe” or overlapping with the “severe” range. Among patients for whom information was available, the median age of patients’ first words (n = 30; 7 non-verbal; 39 unknown) was markedly delayed to 30 months (IQR = 18.75–36) and their first steps/ability to walk (n = 28; 48 unknown) was mildly delayed to 15 months (IQR = 13.13–18.13). Among those with reported behavioral abnormalities, aggression (7/21, 33.33%) was among those most frequently reported, aside from ASD-like and ADHD-like features.

A few patients experienced regression (8/55, 14.55%), primarily of expressive language. Psychiatric diagnoses were less commonly reported (7/53, 13.21%), with anxiety most frequent (5/54, 9.26%, OMIM:607834). Other neurological complications included sleep abnormalities (16/56, 28.57%), coordination abnormalities or gait disturbances (12/53, 22.64%), hypotonia (12/54, 22.22%), seizures (8/56, 14.29%), and toe walking (6/53, 11.32%). Most seizures manifested as generalized (7/8, 87.5%), 4 were uncomplicated febrile seizures, and all onset at ≤7 years old or age was otherwise not specified.

The most frequently observed non-developmental features included ophthalmological conditions (38/58, 65.52%) – most frequently, myopia (29/54, 53.70%), strabismus (16/56, 28.57%, OMIM:185100), and astigmatism (6/52, 11.54%, OMIM:603047) – and musculoskeletal/orthopedic abnormalities (30/57, 52.63%) – most frequently abnormal thorax morphology (8/53, 15.09%), clinodactyly (5/54, 9.26%), Achilles tendon contractures (4/53, 7.55%), bilateral club feet (4/54, 7.41%), and pes planus (3/53, 5.66%). Three cases were described with insulin resistance/pre-diabetes (n = 1) or diabetes (type 1, n = 1, OMIM:222100; type 2, n = 1, OMIM:125853). Other notable conditions included respiratory/ENT abnormalities (18/55, 32.73%) – including chronic otitis media infections (12/54, 22.22%, OMIM:166760). Hearing deficits were described (8/52, 15.38%), many of which were conductive or due to a history of chronic infections. Abnormal brain imaging was reported in 10/27 (37.04%) patients with available magnetic resonance imaging (MRI). A subset of 4 patients demonstrated periventricular abnormalities on MRI, including periventricular hyperintensities, white matter volume loss, and/or leukomalacia. Two of these cases were described as having signs of upper motor neuron dysfunction and spastic paraplegia. While two had unremarkable pregnancies/deliveries, one was born prematurely and one was complicated by perinatal asphyxia.

Dysmorphic craniofacial features were frequent (44/60, 73.33%); however, specific features varied and were not uniformly observed. The most frequently reported features included abnormalities of ear morphology (13/59, 22.03%), hypertelorism and/or telecanthus (12/58, 20.69%, OMIM:145400), down-slanted palpebral fissures (11/58, 18.97%), as well as frontal bossing and/or prominent forehead (9/58, 15.52%).

Patients with shared genetic variants (n = 7 variants among 14 patients) had similar neurodevelopmental presentations; however, some features, such as craniofacial dysmorphic features and orthopedic abnormalities, were variably present, suggesting potential differences in penetrance of certain phenotypes and/or variable expression. For example, unrelated patients P11 and P18 (NM_001367943.1:c.162del, NP_001354872.1:p.(Asn55IlefsTer53), NG_012631.1:g.5669del, NC_000010.11:g.112950918del) both presented with speech delay, gross motor delay, concentration deficiency (no formal diagnosis of ADD/ADHD), behavioral issues, and myopia. However, P11 additionally presented with craniofacial dysmorphic features, orthopedic abnormalities (including abnormal thorax morphology), hypotonia, and absence of facial/axillary hair discordant with age. P18, by contrast, presented with a growth disorder with delayed bone age, primary enuresis, strabismus, no craniofacial dysmorphic features, and no orthopedic abnormalities.

Other patients with shared variants, P8 and P48 (NM_001367943.1:c.1172T>C, NP_001354872.1:p.(Leu391Pro), NG_012631.1:g.207094T>C, NC_000010.11:g.113152343T>C), had speech delay, concentration deficiency or diagnosed ADHD, normal initial motor development, myopia, orthopedics abnormalities (lumbar hyperlordosis vs. brachydactyly and syndactyly), and recurring ear infections and chronic otitis media. However, P48 had no neurologic features or craniofacial dysmorphic features, while P8 additionally presented with ASD, limb hypertonia and CN IV paresis, as well as craniofacial dysmorphic features (hypertelorism and downslanted palpebral fissures among others).

Compared to patients first identified in Dias et al.^30^ (n = 12), remaining cases in this cohort (n = 64) had no statistically significant differences across all HPO-standardized terms and categories of phenotypic features. There was a nominally increased frequency of ASD in our cohort (69.57% vs. 33.33% Dias; nominal p-value = 0.042) and decreased frequency of hypertrichosis (0% vs. 22.2% Dias; nominal p-value = 0.029). However, these differences did not survive correction for multiple hypothesis testing (Figure 3C, Supplemental Figure 2).

### TRND patient phenotypes do not grossly cluster by genomic locus or transcript domain

Next, we sought to investigate if variant type and genomic locus were associated with phenotypic differences among patients. A comparison between variant types is displayed in Figure 3D and Supplemental Figure 3. When compared to non-CNV variant groups, patients with CNV variants exhibited higher frequencies of cardiac abnormalities compared to patients with missense (100.0% vs. 9.09% missense; BH-adjusted p-value = 0.029) and out-of-frame indel variants (100.0% vs. 7.14% out-of-frame indel; BH-adjusted p-value = 0.029). This difference was of nominal significance when compared to other variant types (splice-site, nonsense) but did not survive multiple hypothesis testing. Other inter-group differences were not significant following Benjamini-Hochberg adjustment, and differences of nominal significance are summarized in Supplemental Figure 3.

Despite the limited sample size, we investigated whether phenotypic differences existed among variants predicted to disrupt all 17 RefSeq isoforms of both FL- and DN-*TCF7L2* (n = 72) vs. FL-*TCF7L2* only (15 isoforms; n = 4) (Figure 3E). Variants predicted to affect the protein sequence of only FL-*TCF7L2* isoforms were more frequently associated with short palpebral fissures (50.0% vs. 3.70%; nominal p-value = 0.021) and skin/hair/nails abnormalities (75.0% vs. 17.65%; nominal p-value = 0.029), including sparse lateral eyebrows (50.0% vs. 0%; nominal p-value = 0.004) and slow-growing hair (50.0% vs. 0%; nominal p-value = 0.004). However, these differences did not survive multiple hypothesis correction. While not statistically significant, 2/4 patients with variants predicted to impact only FL-*TCF7L2* transcripts had notable endocrine abnormalities (50.0% vs. 10.9%; nominal p-value = 0.09), including a growth disorder with delayed bone age and absent axillary/facial hair in adolescence. Differences between the two isoform groups – across all HPO-standardized phenotypes – are summarized in Supplemental Figure 4.

Next, we sought to evaluate if clinical presentation varied based on variant location within the *TCF7L2* gene (Supplemental Figure 5). Generally, specific phenotypes across patients were well-distributed in type and location across the *TCF7L2* transcript but a few trends were noted. Much of the sub-phenotypes of orthopedics/musculoskeletal abnormalities were concentrated beyond exon 6. Notably, severe phenotypes, including seizures, did not grossly cluster with location on the *TCF7L2* transcript but were often sequestered between exons 5 and 11 (Supplemental Figure 5). A subset of patients with variants clustering at the terminal end of the HMG Box domain presented with co-occurring craniofacial features, including frontal bossing, downslanted palpebral fissures, and ear morphology abnormalities.

### Small retrospective adult cohort with pLOF TCF7L2 variants nominally associated with increased risk of T2D

To explore phenotypes in adults with pLOF *TCF7L2* variants, we conducted a retrospective analysis of PMBB adults. Following enforcement of inclusion and exclusion criteria (Methods), 11 patients were identified – of whom 2 had nonsense, 7 splice-site, and 2 out-of-frame indel variants introducing a premature stop codon (Supplemental Table 5). All had 2 or more EHR clinician encounters documented. However, notably, none of the variants impacted all *TCF7L2* isoforms. In fact, 9/11 impacted only one FL-*TCF7L2* isoform. Notably, a neurodevelopmental phenotype could not be confirmed in any PMBB patients in this cohort (n = 11) potentially due to a lack of a neurodevelopmental phenotypes or limitations of this methodology and represents a distinct group compared to “Likely TRND” patients. The median age of this cohort was 55.06 years (IQR = 54.0–74.5), 7/11 (63.6%) were male, and median BMI was 31.75 (IQR = 26.78–35.44). Comparatively, the median age of the control cohort (n = 198) was 60.11 years old (p-value = 0.3944), 119/198 (60.1%) were male (p-value = 1), and median BMI was 28.60 (IQR = 24.85–32.61; p-value = 0.1539). The lack of neurodevelopmental phecodes, and inability to confirm a neurodevelopmental phenotype, limit the ability to make clear inferences from this cohort to TRND patients. PMBB patients did trend toward a marginally increased risk of some features identified from the “Likely TRND” cohort, including congenital deformities of the limbs/feet (OR = 9.8; nominal p-value = 0.15) and myopia (OR = 1.1375; nominal p-value = ~1) in the absence of increased risk of other congenital anomalies; however, these associations were not statistically significant.

Across all 1748 distributed phecodes, T2D with renal manifestations (OR = 5.81; 3/11 vs. 12/198; nominal p-value = 0.03) and chronic kidney disease (OR = 8.91; 3/11 vs. 8/198; nominal p-value = 0.01), were both represented at increased frequency among PMBB patients meeting pLOF *TCF7L2* variant criteria. *TCF7L2* has been historically associated with metabolic disorders; metabolic syndromes, including dysmetabolic syndrome X, were trending towards an association with patients in the PMBB cohort (OR = 6.5; nominal p-value = 0.196), but the difference was not statistically significant. Some non-metabolic pathologies were nominally associated with the PMBB patient cohort compared to controls, including unspecified diseases of hair and hair follicles (OR = 8.58; nominal p-value = 0.046). However, no differences survived multiple hypothesis testing. Neurodevelopmental and other phenotypic comparisons between the “Likely TRND” and PMBB pLOF TCF7L2 variant cohorts were not performed due to lack of overlapping phecodes, including age-relevant diagnoses. A composite summary of odds ratios and p-values for the full list of phecodes is provided in Supplemental Table 6.

## Discussion

This study characterizes the largest cohort of patients to date with *TCF7L2* pLOF variants constituting *T**CF7L2*-related neurodevelopmental disorder, or TRND. There are several key findings arising from this study.

First, the spectrum of *TCF7L2* genetic variants implicated in TRND includes missense, nonsense, indel, and splice region variants. CNV variants did not differ phenotypically with the exception of cardiovascular abnormalities. Consistent with Dias et al.^20^, a number of missense variants localized to the C-terminal ends of the HMG Box and CTNNB1 domains, though, *de novo* variants were also identified between the CTNNB1 and HMG Box domains and upstream of Exon 4. This study identifies *TCF7L2* variants before Exon 4 and could yield insight into functional differences for FL- vs. DN-*TCF7L2* transcripts. Variants without predicted effects on the coding sequence of DN-*TCF7L2* transcripts were associated with differences in vision/ophthalmological, endocrine, and skin/hair/nails manifestations – though differences were not statistically significant and caution should be used in interpretation of this result. Additional cases are warranted, and if these differences are re-observed with greater sample size, one hypothesis is that impacting DN-*TCF7L2* transcripts contributes to these differences. DN-*TCF7L2* has unique patterns of selective tissue expression (e.g., DN-*TCF7L2* expression has been detected in murine brain but not murine liver or pancreas), which can be postulated to contribute to these phenotypic differences^38–39^.

Second, across this expanded cohort, neurodevelopmental, visual, and orthopedic/musculoskeletal abnormalities were most prevalent and likely form the defining syndromic features of TRND (Figure 3A, Supplemental Figure 1). Craniofacial dysmorphism was observed in a majority of patients (44/60, 73.33%), with heterogeneous features – most commonly abnormal ear morphology, hypertelorism, down-slanted palpebral fissures, as well as frontal bossing and/or a prominent forehead. These findings align with Dias et al.^20^, though a nominally higher frequency of ASD and under-representation of hypertrichosis was observed; however, the Dias cohort was small (n = 12) and this did not survive multiple hypothesis correction. Increased ASD representation may be attributed to selection bias as ASD repositories and articles were searched to find patients in this study. Importantly, this study expands on and establishes a spectrum of sub-phenotypes across phenotypic categories. Abnormal thorax morphology, Achilles tendon contractures, and clinodactyly were the most prevalent orthopedic/musculoskeletal abnormalities while myopia, strabismus, and astigmatism were the most frequently represented ophthalmologic abnormalities. Phenotypic differences by isoform or variant type of nominal significance did not survive multiple hypothesis testing and should be interpreted with caution. Phenotypic differences also did not grossly cluster by genomic locus. Interestingly, some patients, such as SP1, had a *de novo* nonsense variant but no reported neurodevelopmental phenotype. Additionally, some patients (e.g., P11/18) shared variants but differed in key phenotypic traits. There are several potential explanations for this, including variable expressivity and/or differences in penetrance, additive contributions of other unknown variants, epigenetic differences, differences due to mode of inheritance (mosaicism and differences in mutational burden for germ-line vs. post-zygotic variants), and/or limitations in data collection (e.g., inter-observer differences) or timing of symptom manifestation. Functional studies on biospecimens from these patients may clarify mechanisms contributing to variable feature manifestation.

Third, this study includes a biobank-based search of thousands of adults for pLOF variants in *TCF7L2*. In a retrospective case-control analysis of PMBB patients, carriers of pLOF *TCF7L2* variants demonstrated a nominally increased risk of T2D with renal manifestations (p-value = 0.03), but this did not survive multiple hypothesis testing and there was a trend towards increased BMI between the cohorts. Expanded searches across additional biobanks could clarify the validity of this association. Due to phecode restraints and the retrospective design, pediatric neurodevelopmental phenotypes (and other indirect phenotypic markers of the “Likely TRND” group) of these adult PMBB patients could not be assessed. In addition to ambiguity added by absence of a reported neurodevelopmental phenotype, interpretation of these findings is further limited by nominal significance and absence of functional evidence supporting loss-of-function. Thus, direct comparisons with the pediatric group and inference of relative risk in TRND are not possible. Neurodevelopmental follow-up of adult PMBB patients and validation of functionally reduced *TCF7L2* expression of patients’ banked samples is required to: (1) correlate with TRND’s natural history; and (2) estimate TRND population incidence.

Since *TCF7L2*’s discovery as a locus strongly associated with T2D^4^, there has been controversial evidence regarding how intronic risk alleles put patients at increased risk for T2D – as a result of agonistic or antagonistic effects on *TCF7L2* expression or non-*TCF7L2*-mediated mechanisms. In the primary “Likely TRND” cohort, only 2 cases of diabetes were identified – one of whom was an adult with T2D – potentially reflecting the patient cohort’s young median age. Future functional investigation of this relationship is needed through patient-derived sources (e.g., induced pluripotent stem cells)^40^.

This study has several limitations. First, uniform characterization of patients was challenging due to heterogeneous identification methods, leading to potential data gaps and increased susceptibility to ascertainment bias. Further, as previously noted, searching ASD repositories may introduce selection bias, increasing representation of ASD. We sought to mitigate both of these risks by implementing HPO-standardized ontology terms across cases and differentiating absence (“0”) of phenotypic features vs. lack of clinical information (“N/A”) to accommodate differences in confidence in age-specific clinical assessment. Making this distinction mitigated the dilution of features that would otherwise be under-represented by inclusion of patients from these repositories. Importantly, no other features were grossly enriched among these patients that would be likely also over-represented with ASD. Secondly, retrospective EHR data analysis has inherent limitations, including incomplete or inaccurate documentation of clinical features, lack of longitudinal data predating tertiary care at PennMedicine, and potential ascertainment/referral bias. The adult cohort further lacks neurodevelopmental phenotyping, limiting direct comparisons with the pediatric TRND group. Thirdly, by nature of this investigation, a lack of functional studies limits validation of some identified variants, and, toward that end, *in vitro* and *in vivo* validation experiments replicating these variants would further corroborate these findings.

In conclusion, this study establishes an expanded genotypic and phenotypic spectrum for TRND that requires further investigation and functional validation. We have launched a natural history study with Simons Searchlight (https://www.simonssearchlight.org/research/what-we-study/tcf7l2/) that is open for enrollment to further characterize TRND patients.

## Supplementary Material

Supplemental Figures S1-S5

Supplemental Tables S1-S6

## Figures and Tables

**Figure 1.**
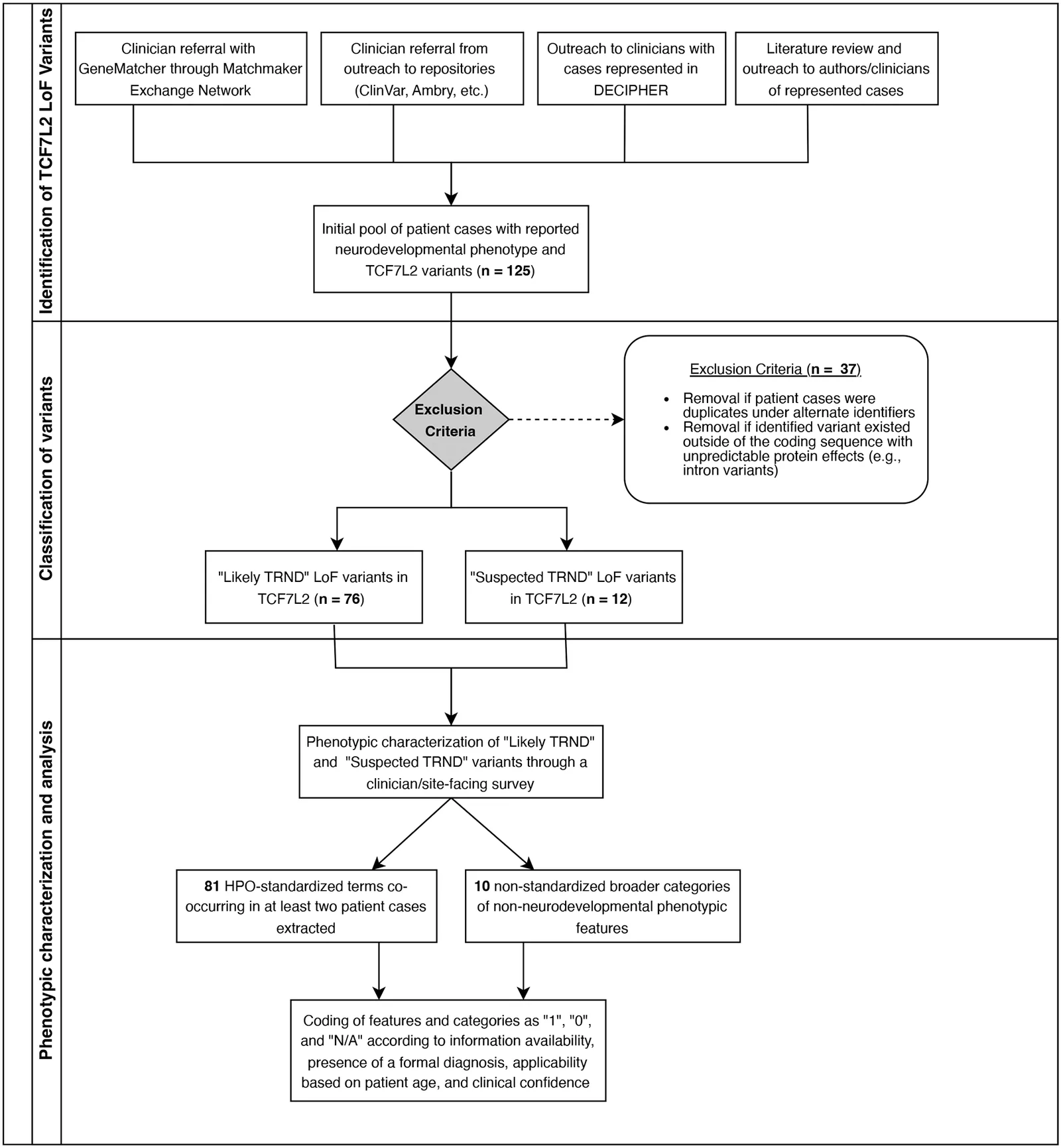
Search, identification, and characterization of TRND patient cases. Orthogonal approaches were adopted to identify and characterize patient cases with predicted loss of function TCF7L2 variants. 76 variants were classified as likely loss-of-function TCF7L2 variants resulting in TCF7L2-related neurodevelopmental disorder (TRND) (Supplemental Table 1). 12 patient cases were suspected to have TRND but did not otherwise meet criteria (Supplemental Table 4). We phenotypically characterized “Likely TRND” and “Suspected TRND” cases using a clinician/site-facing tabular survey with free-response questions. Of the 76 “Likely TRND” and 12 “Suspected TRND” patients, 20 and 7, could not be further characterized with additional information through the tabular survey by the referring clinician/site/author. For analytical purposes, we extracted Human Phenotype Ontology (HPO)-standardized terms that co-occurred in at least two patient cases, comprising 81 features, and 10 categories of non-HPO broader categories of phenotypic features. For the purposes of aggregated counts, patients were assigned a “1” for a phenotypic feature if a qualified healthcare professional reported its presence in the survey on clinical assessment or through patient report. “0” was designated in the absence of the phenotypic feature on clinical assessment or through report, and “N/A” was assigned if phenotypic information was unavailable or could not otherwise be definitively determined or clinically assessed -- altering the denominator per phenotypic feature or category.

**Figure 2.**
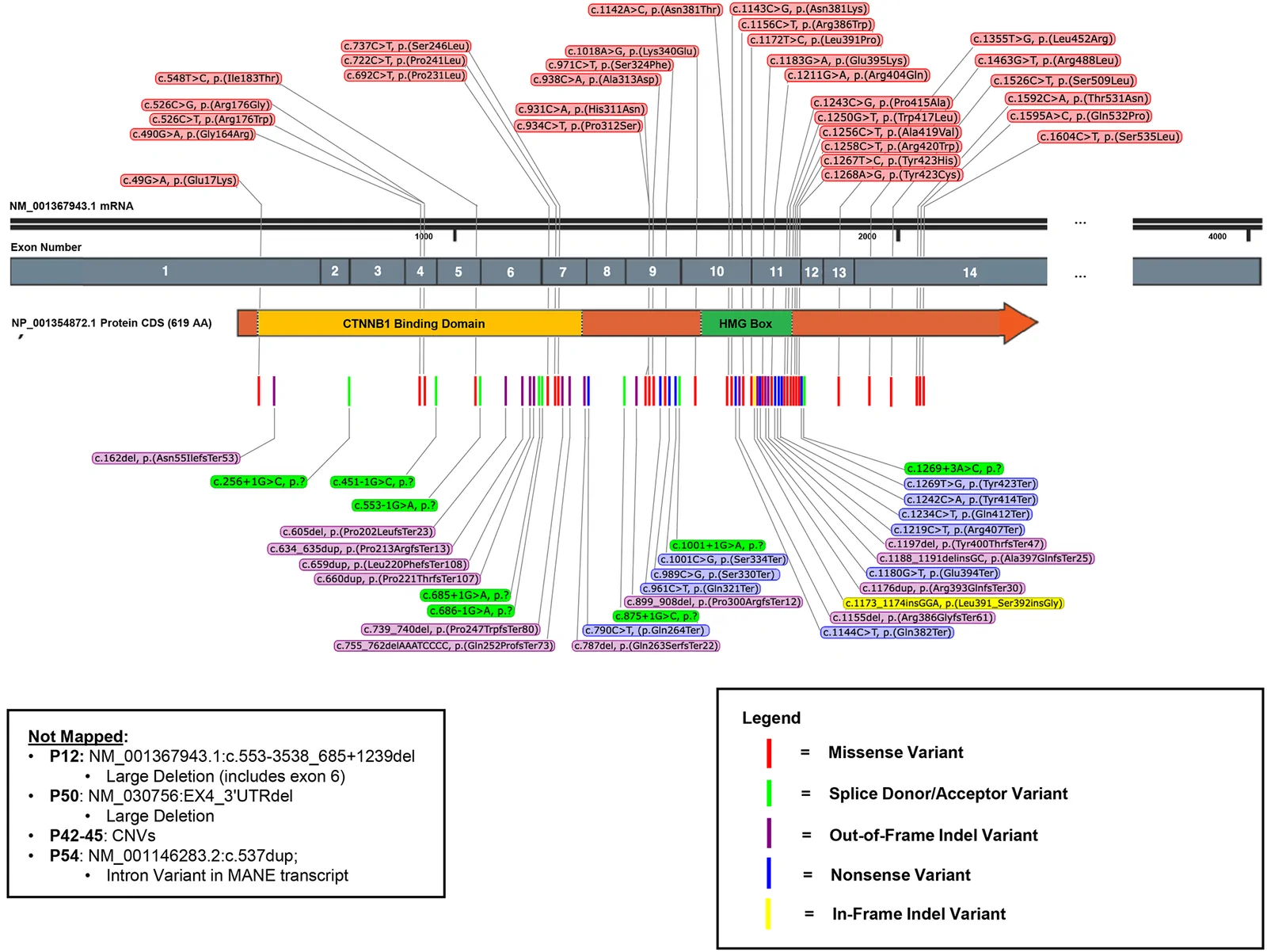
Gene map of patients with likely TCF7L2-related neurodevelopmental disorder (TRND). The spectrum of genetic variants represented in “Likely TRND” patient cases are mapped to the *TCF7L2* MANE select transcript (NM_001367943.1) and its associated protein product (NP_001354872.1). Variants are distributed across highly conserved regions of the *TCF7L2* genes, including foci within the CTNNB1 Binding, the HMG Box domains, as well as in a region between them.

**Figure 3.**
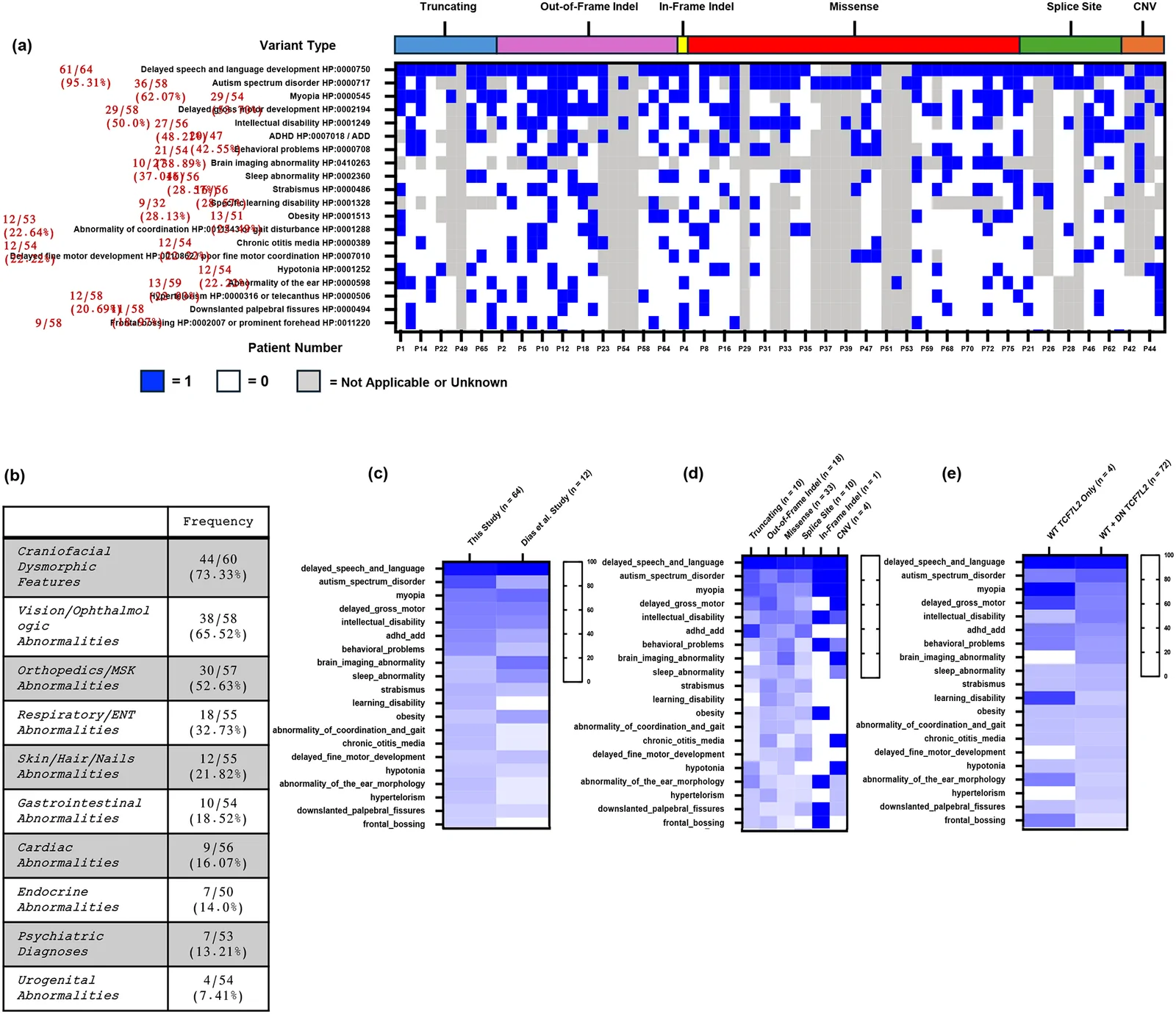
Top 25% highest frequency phenotypic attributes occurring in TRND patient cases. 81 HPO-standardized phenotypic terms co-occurring in two or more “Likely TRND” patients were extracted as well as 10 non-standardized broader categories of non-neurodevelopmental features. (**a**) The top 25% highest frequency HPO-standardized phenotypes among “Likely TRND” patients are grouped by variant type. While the top 25% highest frequency phenotypic attributes are included, the full distribution is included in Supplemental Figure 1. The highest frequency HPO terms among “Likely TRND” patient cases included speech delay HP:0000750 (95.31%), autism spectrum disorder HP:0000717 (62.07%), myopia HP:0000545 (53.70%), delayed gross motor development HP:0002194 (50.0%), and intellectual disability HP:0001249 (48.21%). (**b**) The highest frequency broader categories of non-neurodevelopmental phenotypic features included craniofacial dysmorphic features (73.33%), vision/ophthalmologic abnormalities (65.52%), and orthopedics/musculoskeletal (MSK) abnormalities (52.63%). For the top 25% highest frequency phenotypic attributes in (a), differences in phenotypic frequency were evaluated across (c) variant type, (d) in comparison with the Dias et al. study^6^ (11 patients from the primary cohort and 1 identified from the Dias et al.^6^ supplement; n = 12), and (e) across isoform types predicted to be disrupted. Comparisons of the full distributions are included in Supplemental Figures 2–4.

**Table 1.** Features of TRND patient cohorts. Patients with predicted loss of function (pLOF) TCF7L2 variants were collected using an orthogonal approach, including GeneMatcher, DECIPHER, individual outreach to referred clinicians, and literature review prior to phenotypic characterization. 76 patient cases were identified with a median age of 8.17, and 44.29% of whom were female. Patients who were suspected to have TCF7L2-related neurodevelopmental disorder but did not meet criteria were classified as “Suspected TRND”.

	Patient Cohort with “Likely TRND” (n = 76)	Patient Cohort with “Suspected TRND” (n = 12)
**Patient Features**		
**Median Age (1**^**st**^ **Quartile, 3**^**rd**^ **Quartile)**^a^	**8.17** (3.94, 13.25)	**13** (6.5, 22.5)
**Sex (% Female)** ^b^	**31/70** (44.29%)	**3/9** (33.33%)
**Ethnicity Distribution** ^c^		
*White Non-Hispanic*	**30** (39.47%)	**4** (33.33%)
*Asian Non-Hispanic*	**2** (2.63%)	**2** (16.67%)
*Black or African American Non-Hispanic*	**2** (2.63%)	**0** (0.0%)
*Hispanic*	**1** (1.32%)	**0** (0.0%)
*Not Known or Prefer Not to Disclose*	**41** (53.95%)	**6** (50.0%)
**Median Height Z-Score (1**^**st**^ **Quartile, 3**^**rd**^ **Quartile)**^d^	**0.14** (−0.95, 0.66)	−**0.45** (−0.66, 0.06)
**Median Weight Z-Score (1**^**st**^ **Quartile, 3**^**rd**^ **Quartile)**^e^	**0.85** (−0.25, 1.60)	**0.23** (−0.14, 0.79)
**Variant Features and Distribution**		
**Variant Class**		
*Nonsense (Stop-Gain)*	**10** (13.16%)	**4** (33.33%)
*Out-of-Frame Indel*	**18** (23.68%)	**1** (8.33%)
*Missense*	**33** (43.42%)	**6** (50.0%)
*Splice Site Donor/Acceptor Variant*	**10** (13.16%)	**1** (8.33%)
*In-Frame Indel*	**1** (1.32%)	**N/A**
*CNV Deletion*	**4** (5.26%)	**N/A**
**Cases with Shared : Unique Variants**	**14:62**	**4:8**
**Inheritance Pattern**		
*De novo* ^f^	**58** (76.32%)	**4** (33.33%)
*Inherited* ^g^	**7** (9.21%)	**4** (33.33%)
*Unknown*	**11** (14.47%)	**4** (33.33%)
**Missense Variant Location**		
*HMG Box Domain*	**12** (36.36%)	**N/A**
*CTNNB1 Binding Domain*	**8** (24.24%)	**2** (33.33%)
*Neither*	**13** (39.39%)	**4** (66.67%)

a Median and quartiles found relative to number of patients with available information (**n = 64** for “Likely TRND”, **n = 6** for “Suspected TRND”)

b Reflective of the number of patients with available information about sex (**n = 70** for “Likely TRND”, **n = 9** for “Suspected TRND”)

c Ethnicities based on categories defined by the Center for Disease Control (CDC). There were no patients of American Indian and Alaskan Native descent or Native Hawaiian or Other Pacific Islanders represented in this cohort.

d Median and quartiles found relative to number of patients with available information (**n = 48** for “Likely TRND”, **n = 4** for “Suspected TRND”)

e Median and quartiles found relative to number of patients with available information (**n = 49** for “Likely TRND”, **n = 4** for “Suspected TRND”)

f De novo or likely de novo (**n = 4** could not confirm absence in both parents)

g Most inherited variants had unknown parental phenotypes (**n = 4**). Others had a parent with mosaicism with school difficulties but no reported neurodevelopmental delays (**n = 2**). **n = 1** patient had a homozygous inherited variant in *TCF7L2* with no neurodevelopmental delays in either parent.

## Data Availability

All genetic and phenotypic data generated from survey collection and during analysis are included in this published article and its supplemental content.

## Appendix

Consortia

UCI GREGoR Site

Robert Daber

Emily Westheimer

William O’Callaghan

Jianhua Zhao

Nirav Malani

Ingrid Chen

Kaylee Scozarro

Michelle Barbosa

Andrea Cohen

Arthur Ko

Emmanuele Delot

Georgia Pitsava

Seth Berger

Lightning Auriga

Ya Cui

Changrui Xiao

Charles Hadley King

Eric Vilain

Ivan de Dios

Jonathan LoTempio

Miguel Almalvez

Vincent Fusaro

Wei Li

Jamie Fraser

Meghan Delaney

Leandros Boukas

Bojan Losic

Esther (Yun-Hua) Hsiao

John Harting

Krista Bluske

Rachid Karam

Sami Belhadj
