# Supplementary material for: Characterization of the genotypic and phenotypic spectrum of TCF7L2-related neurodevelopmental disorder (TRND)
Source: Genet Med. Author manuscript; Available in PMC 2026 Oct 24. (PMC13693294; doi:10.1016/j.gim.2026.102642)
Supplement: Supplementary file 1 [file NIHMS2217154-supplement-Supplemental_Figures_S1-S5.pdf]

Supplemental Figure 1. Phenotypic attributes co-occurring in TRND patient cases. A full display of all HPO-standardized terms co-occurring in at least two TRND cases, along with their distribution across "likely TRND" patient cases, is included. 81 HPO-standardized terms were identified (included below), in addition to 10 broad categories of non-neurodevelopmental phenotypic features.

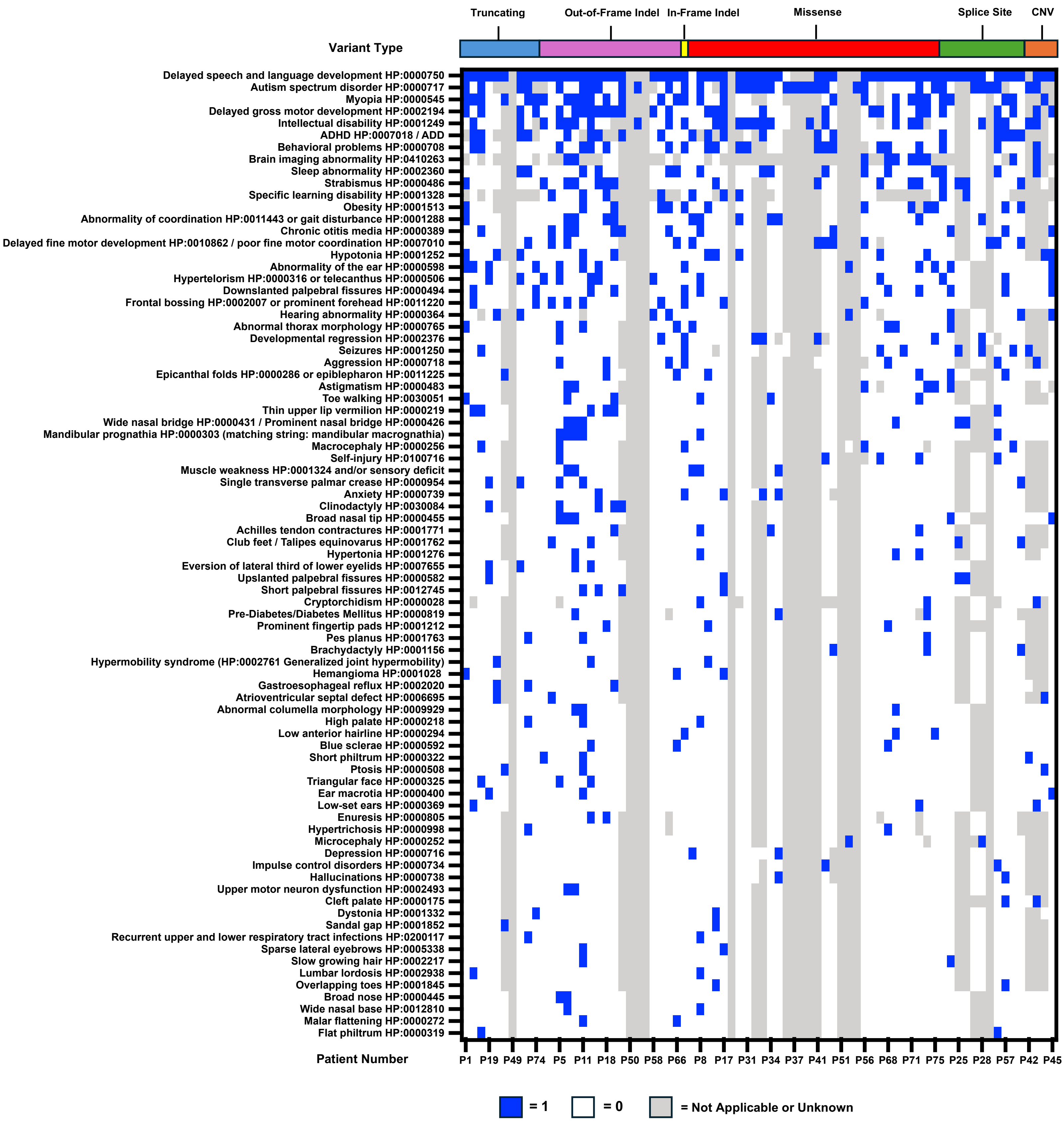

Supplemental Figure 2. HPO-standardized phenotypic comparisons with Dias et al. Differences between patients in this study and those originally explored by Dias et al. were evaluated across 81 HPO-standardized terms (included below). There was a nominally increased frequency of ASD in our cohort (69.57% vs. 33.33% Dias et al.; un-adjusted p-value: 0.042) and a decreased reported frequency of hypertrichosis (0% vs. 22.2% Dias et al.; un-adjusted p-value: 0.029). However, these differences did not survive correction for multiple hypothesis testing, and other observed differences were not statistically significant.

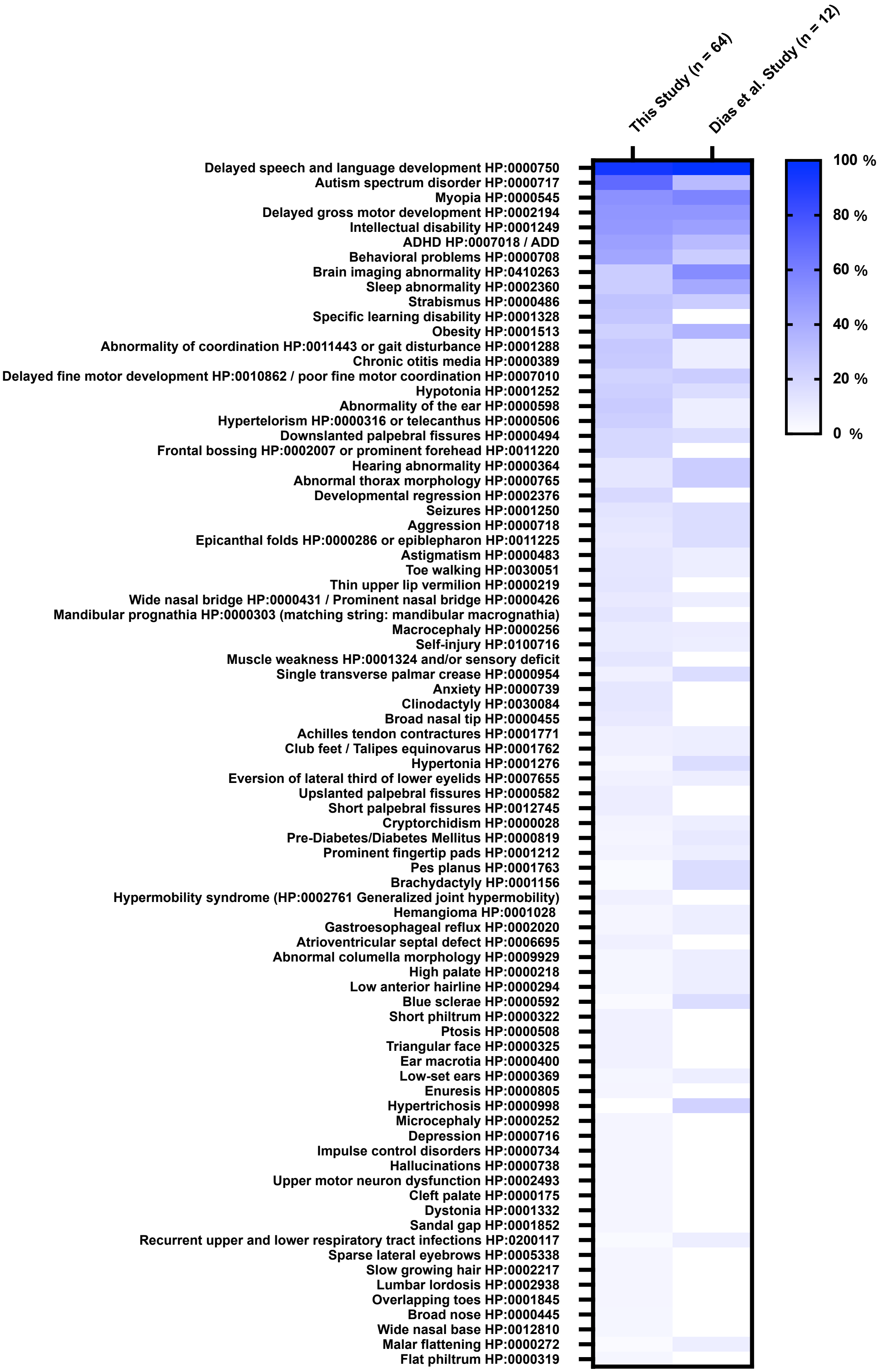

Supplemental Figure 3. HPO-standardized phenotypic comparisons by variant type. Differences between variant type groups (truncating, out-of-frame indels, missense, splice site, in-frame indels, and CNV variants) were evaluated across 81 HPO-standardized terms (included below). When compared to non-CNV variant groups, patients with CNV variants exhibited higher frequencies of cardiac abnormalities compared to patients with missense and out-of-frame indel variants (100.0% vs. 9.09% missense; BH-adjusted p-value: 0.029; 100.0% vs. 7.14% out-of-frame indel; BH-adjusted p-value: 0.029). This difference was nominally significant when compared to other variant types (splice-site, truncating) but did not survive multiple hypothesis testing. Across features and categories, other nominally significant differences included: missense vs. out-of-frame indel: chronic otitis media (9.09% vs. 40%; p-value: 0.042); missense vs. out-of-frame indel: clinodactyly (0% vs. 26.67% ; p-value: 0.021); missense vs. out-of-frame indel: respiratory/ENT abnormalities (18.18% vs. 53.33%; p-value: 0.036); CNV vs. splice site: hypotonia (100.0 vs. 0%; p-value: 0.028); and out-of-frame indel vs. splice-site: seizures (0% vs. 37.5%; p-value: 0.028). These differences did not survive multiple hypothesis testing. Other differences were not statistically significant.

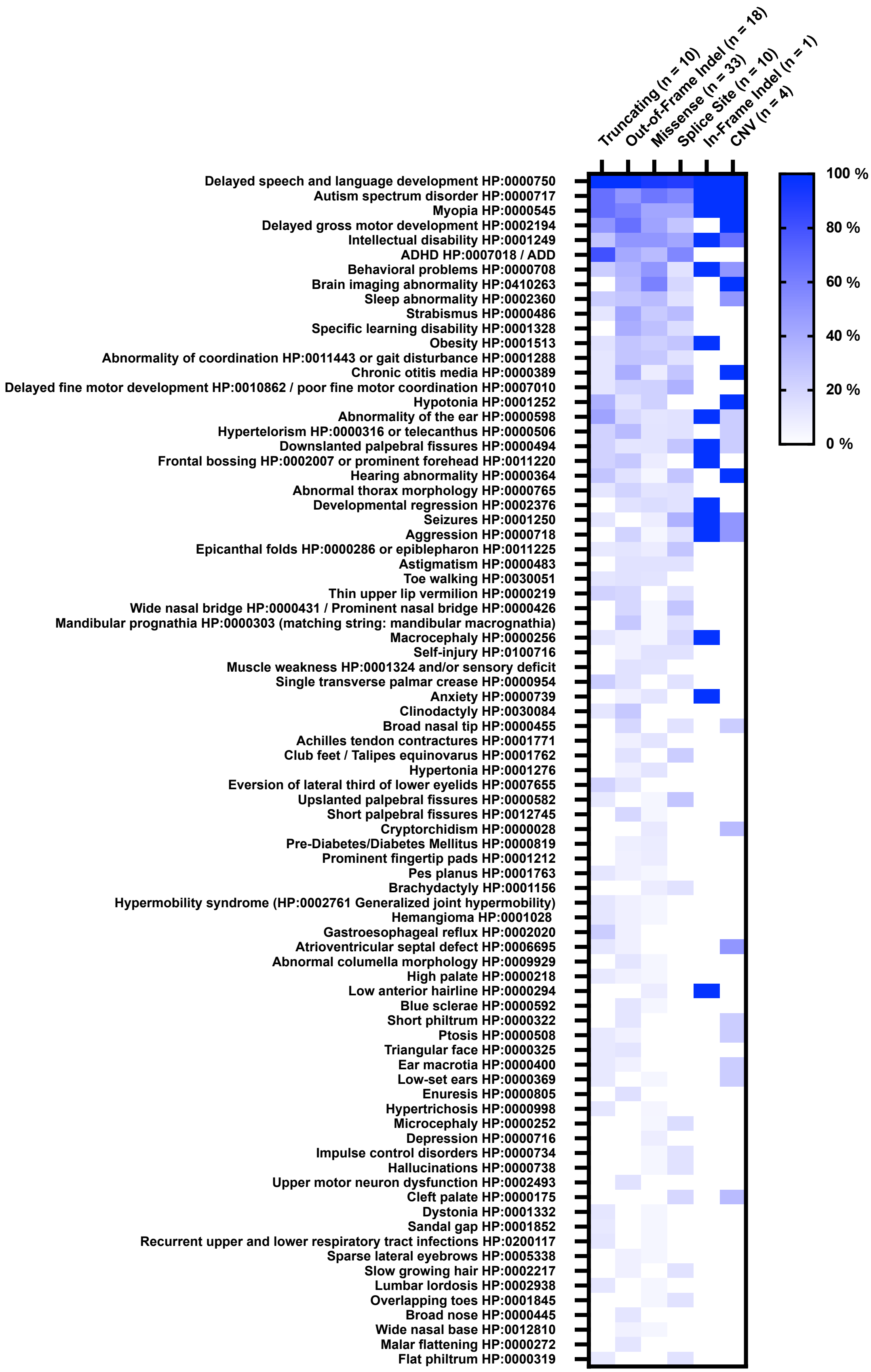

Supplemental Figure 4. HPO-standardized phenotypic comparisons across isoform types. Phenotypic differences between patients with variants predicted to disrupt only wildtype (WT) *TCF7L2* transcript isoforms and those with variants predicted to disrupt both WT and dominant negative (DN) *TCF7L2* transcript isoforms were evaluated across 81 HPO-standardized terms (included below). Variants predicted to functionally affect the protein sequence of only WT *TCF7L2* isoforms were more frequently reported in association with short palpebral fissures (50.0% vs. 3.70%; un-adjusted p-value: 0.021) and skin/hair/nails abnormalities (75.0% vs. 17.65%; un-adjusted p-value: 0.029), including sparse lateral eyebrows (50.0% vs. 0%; un-adjusted p-value: 0.004) and slow-growing hair (50.0% vs. 0%; un-adjusted p-value: 0.004). However, these differences did not survive multiple hypothesis correction, and other differences were not statistically significant.

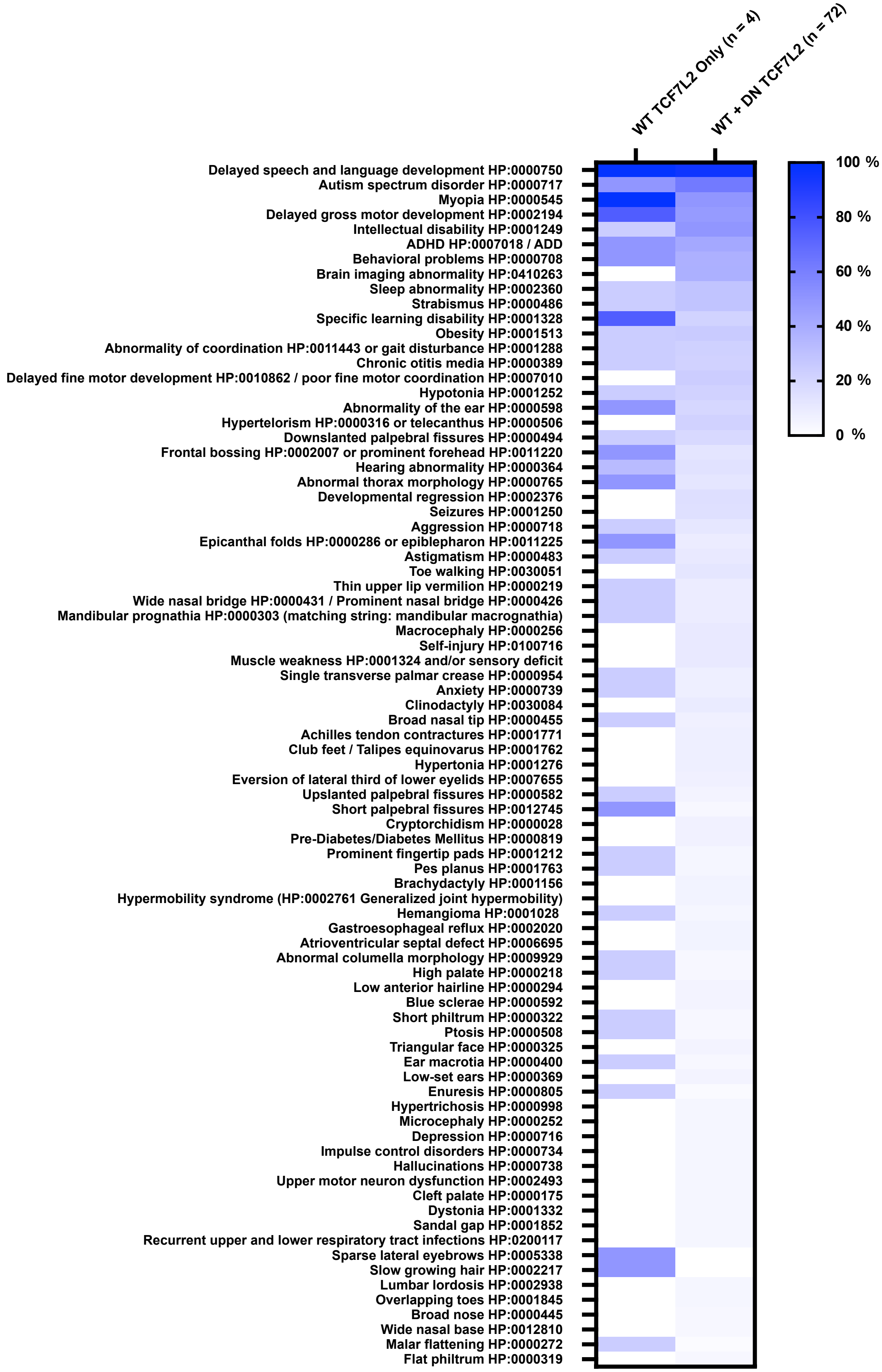

Supplemental Figure 5. Genotype-phenotype correlations of key phenotypic traits in TRND patients. Phenotypes of “Likely TRND” cases were mapped to locations on isoform NM_001367943.1 (MANE transcript). Patient variants unable to be mapped to this isoform are not included below. The absence of a colored bar does not necessarily indicate absence of the feature for that genetic variant, as clinical assessment may be unknown/limited or the variant cannot be mapped (e.g., CNV).

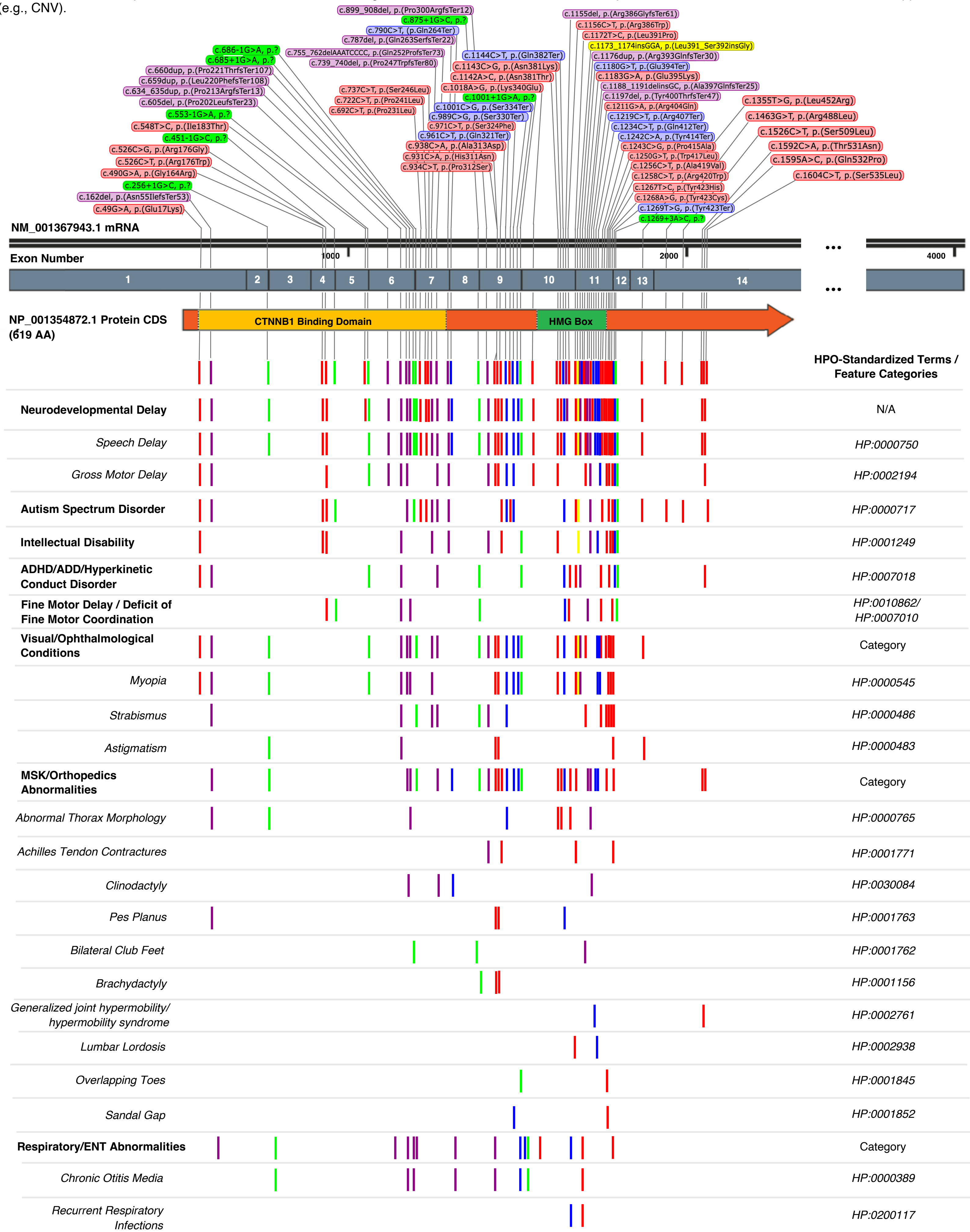

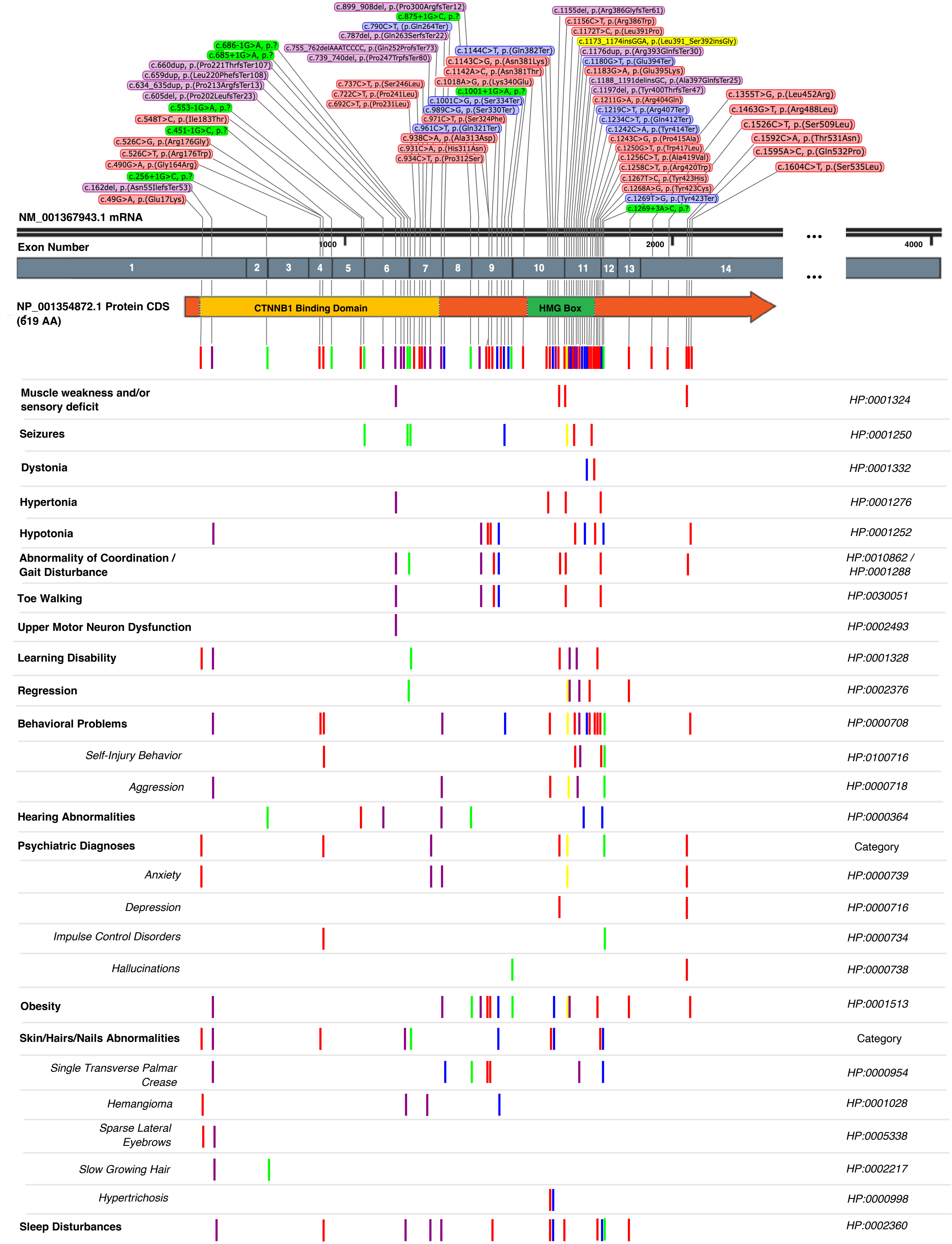

c.899_908del, p.(Pro300ArgfsTer12)
c.875+1G>C, p.?
c.790C>T, (p.Gln264Ter)
c.787del, p.(Gln263SerfsTer22)
c.686-1G>A, p.?
c.685+1G>A, p.?
c.755_762delAAATCCCC, p.(Gln252ProfsTer73)
c.739_740del, p.(Pro247TrpfsTer80)
c.660dup, p.(Pro221ThrfsTer107)
c.659dup, p.(Leu220PhefsTer108)
c.634_635dup, p.(Pro213ArgfsTer13)
c.605del, p.(Pro202LeufsTer23)
c.553-1G>A, p.?
c.548T>C, p.(Ile183Thr)
c.451-1G>C, p.?
c.526C>G, p.(Arg176Gly)
c.526C>T, p.(Arg176Trp)
c.490G>A, p.(Gly164Arg)
c.256+1G>C, p.?
c.162del, p.(Asn55IlefsTer53)
c.49G>A, p.(Glu17Lys)
c.737C>T, p.(Ser246Leu)
c.722C>T, p.(Pro241Leu)
c.692C>T, p.(Pro231Leu)
c.1144C>T, p.(Gln382Ter)
c.1143C>G, p.(Asn381Lys)
c.1142A>C, p.(Asn381Thr)
c.1018A>G, p.(Lys340Glu)
c.1001+1G>A, p.?
c.1001C>G, p.(Ser334Ter)
c.989C>G, p.(Ser330Ter)
c.971C>T, p.(Ser324Phe)
c.961C>T, p.(Gln321Ter)
c.938C>A, p.(Ala313Asp)
c.931C>A, p.(His311Asn)
c.934C>T, p.(Pro312Ser)
c.1155del, p.(Arg386GlyfsTer61)
c.1156C>T, p.(Arg386Trp)
c.1172T>C, p.(Leu391Pro)
c.1173_1174insGGA, p.(Leu391_Ser392insGly)
c.1176dup, p.(Arg393GlnfsTer30)
c.1180G>T, p.(Glu394Ter)
c.1183G>A, p.(Glu395Lys)
c.1188_1191delinsGC, p.(Ala397GlnfsTer25)
c.1197del, p.(Tyr400ThrfsTer47)
c.1211G>A, p.(Arg404Gln)
c.1219C>T, p.(Arg407Ter)
c.1234C>T, p.(Gln412Ter)
c.1242C>A, p.(Tyr414Ter)
c.1243C>G, p.(Pro415Ala)
c.1250G>T, p.(Trp417Leu)
c.1256C>T, p.(Ala419Val)
c.1258C>T, p.(Arg420Trp)
c.1267T>C, p.(Tyr423His)
c.1268A>G, p.(Tyr423Cys)
c.1269T>G, p.(Tyr423Ter)
c.1269+3A>C, p.?
c.1355T>G, p.(Leu452Arg)
c.1463G>T, p.(Arg488Leu)
c.1526C>T, p.(Ser509Leu)
c.1592C>A, p.(Thr531Asn)
c.1595A>C, p.(Gln532Pro)
c.1604C>T, p.(Ser535Leu)
NM_001367943.1 mRNA
1000
2000
4000
Exon Number
1
2
3
4
5
6
7
8
9
10
11
12
13
14
NP_001354872.1 Protein CDS
(619 AA)
CTNNB1 Binding Domain
HMG Box
Muscle weakness and/or sensory deficit
HP:0001324
Seizures
HP:0001250
Dystonia
HP:0001332
Hypertonia
HP:0001276
Hypotonia
HP:0001252
Abnormality of Coordination / Gait Disturbance
HP:0010862 / HP:0001288
Toe Walking
HP:0030051
Upper Motor Neuron Dysfunction
HP:0002493
Learning Disability
HP:0001328
Regression
HP:0002376
Behavioral Problems
HP:0000708
Self-Injury Behavior
HP:0100716
Aggression
HP:0000718
Hearing Abnormalities
HP:0000364
Psychiatric Diagnoses
Category
Anxiety
HP:0000739
Depression
HP:0000716
Impulse Control Disorders
HP:0000734
Hallucinations
HP:0000738
Obesity
HP:0001513
Skin/Hairs/Nails Abnormalities
Category
Single Transverse Palmar Crease
HP:0000954
Hemangioma
HP:0001028
Sparse Lateral Eyebrows
HP:0005338
Slow Growing Hair
HP:0002217
Hypertrichosis
HP:0000998
Sleep Disturbances
HP:0002360

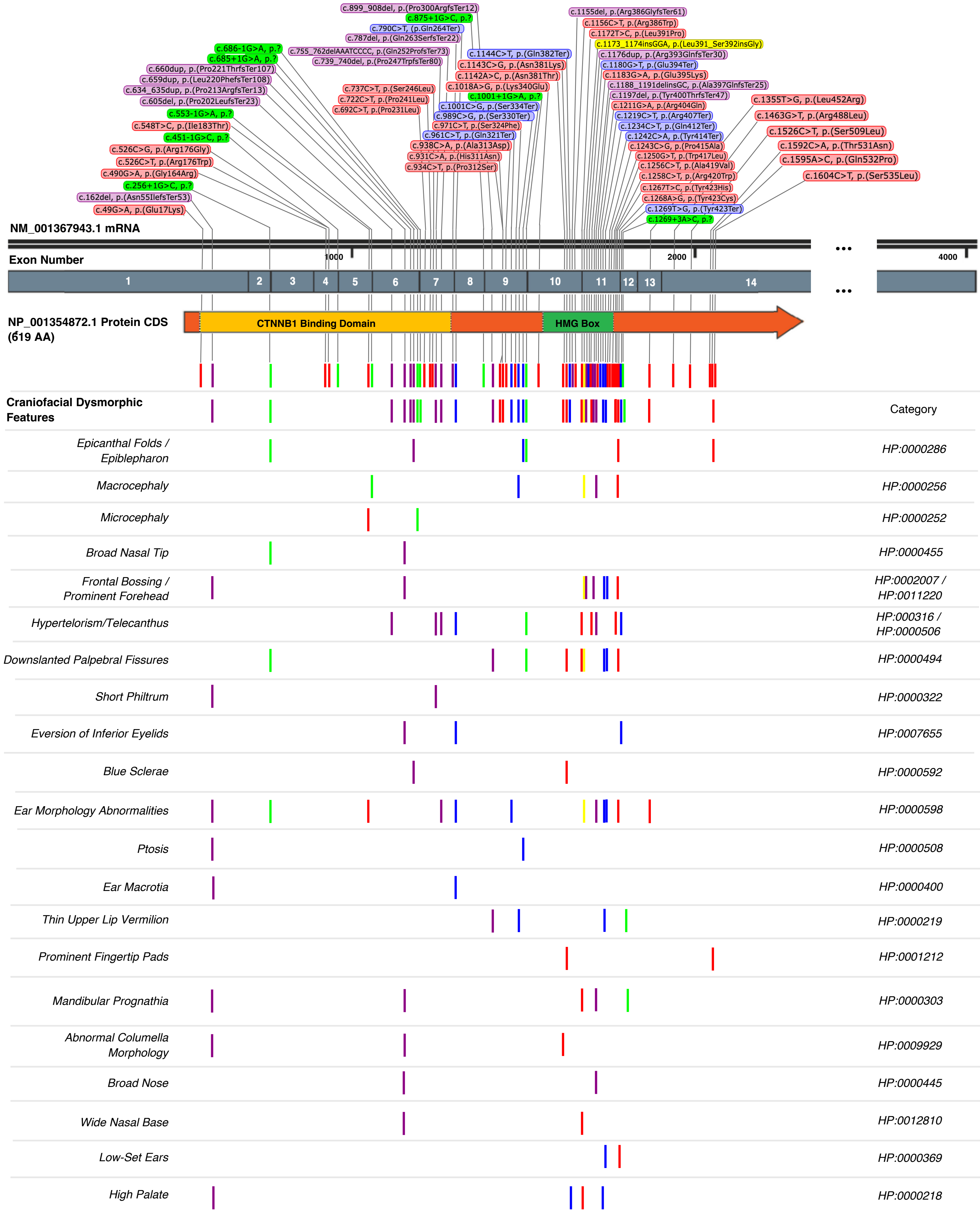
c.899_908del, p.(Pro300ArgfsTer12)
c.875+1G>C, p.?
c.790C>T, (p.Gln264Ter)
c.787del, p.(Gln263SerfsTer22)
c.755_762delAAATCCCC, p.(Gln252ProfsTer73)
c.739_740del, p.(Pro247TrpfsTer80)
c.686-1G>A, p.?
c.685+1G>A, p.?
c.660dup, p.(Pro221ThrfsTer107)
c.659dup, p.(Leu220PhefsTer108)
c.634_635dup, p.(Pro213ArgfsTer13)
c.605del, p.(Pro202LeufsTer23)
c.553-1G>A, p.?
c.548T>C, p.(Ile183Thr)
c.451-1G>C, p.?
c.526C>G, p.(Arg176Gly)
c.526C>T, p.(Arg176Trp)
c.490G>A, p.(Gly164Arg)
c.256+1G>C, p.?
c.162del, p.(Asn55IlefsTer53)
c.49G>A, p.(Glu17Lys)
c.737C>T, p.(Ser246Leu)
c.722C>T, p.(Pro241Leu)
c.692C>T, p.(Pro231Leu)
c.1144C>T, p.(Gln382Ter)
c.1143C>G, p.(Asn381Lys)
c.1142A>C, p.(Asn381Thr)
c.1018A>G, p.(Lys340Glu)
c.1001+1G>A, p.?
c.1001C>G, p.(Ser334Ter)
c.989C>G, p.(Ser330Ter)
c.971C>T, p.(Ser324Phe)
c.961C>T, p.(Gln321Ter)
c.938C>A, p.(Ala313Asp)
c.931C>A, p.(His311Asn)
c.934C>T, p.(Pro312Ser)
c.1155del, p.(Arg386GlyfsTer61)
c.1156C>T, p.(Arg386Trp)
c.1172T>C, p.(Leu391Pro)
c.1173_1174insGGA, p.(Leu391_Ser392insGly)
c.1176dup, p.(Arg393GlnfsTer30)
c.1180G>T, p.(Glu394Ter)
c.1183G>A, p.(Glu395Lys)
c.1188_1191delinsGC, p.(Ala397GlnfsTer25)
c.1197del, p.(Tyr400ThrfsTer47)
c.1211G>A, p.(Arg404Gln)
c.1219C>T, p.(Arg407Ter)
c.1234C>T, p.(Gln412Ter)
c.1242C>A, p.(Tyr414Ter)
c.1243C>G, p.(Pro415Ala)
c.1250G>T, p.(Trp417Leu)
c.1256C>T, p.(Ala419Val)
c.1258C>T, p.(Arg420Trp)
c.1267T>C, p.(Tyr423His)
c.1268A>G, p.(Tyr423Cys)
c.1269T>G, p.(Tyr423Ter)
c.1269+3A>C, p.?
c.1355T>G, p.(Leu452Arg)
c.1463G>T, p.(Arg488Leu)
c.1526C>T, p.(Ser509Leu)
c.1592C>A, p.(Thr531Asn)
c.1595A>C, p.(Gln532Pro)
c.1604C>T, p.(Ser535Leu)
NM_001367943.1 mRNA
1000
2000
4000
Exon Number
1 2 3 4 5 6 7 8 9 10 11 12 13 14
NP_001354872.1 Protein CDS
(619 AA)
CTNNB1 Binding Domain
HMG Box
Craniofacial Dysmorphic Features
Category
Epicanthal Folds / Epiblepharon
HP:0000286
Macrocephaly
HP:0000256
Microcephaly
HP:0000252
Broad Nasal Tip
HP:0000455
Frontal Bossing / Prominent Forehead
HP:0002007 / HP:0011220
Hypertelorism/Telecanthus
HP:000316 / HP:0000506
Downslanted Palpebral Fissures
HP:0000494
Short Philtrum
HP:0000322
Eversion of Inferior Eyelids
HP:0007655
Blue Sclerae
HP:0000592
Ear Morphology Abnormalities
HP:0000598
Ptosis
HP:0000508
Ear Macrotia
HP:0000400
Thin Upper Lip Vermilion
HP:0000219
Prominent Fingertip Pads
HP:0001212
Mandibular Prognathia
HP:0000303
Abnormal Columella Morphology
HP:0009929
Broad Nose
HP:0000445
Wide Nasal Base
HP:0012810
Low-Set Ears
HP:0000369
High Palate
HP:0000218

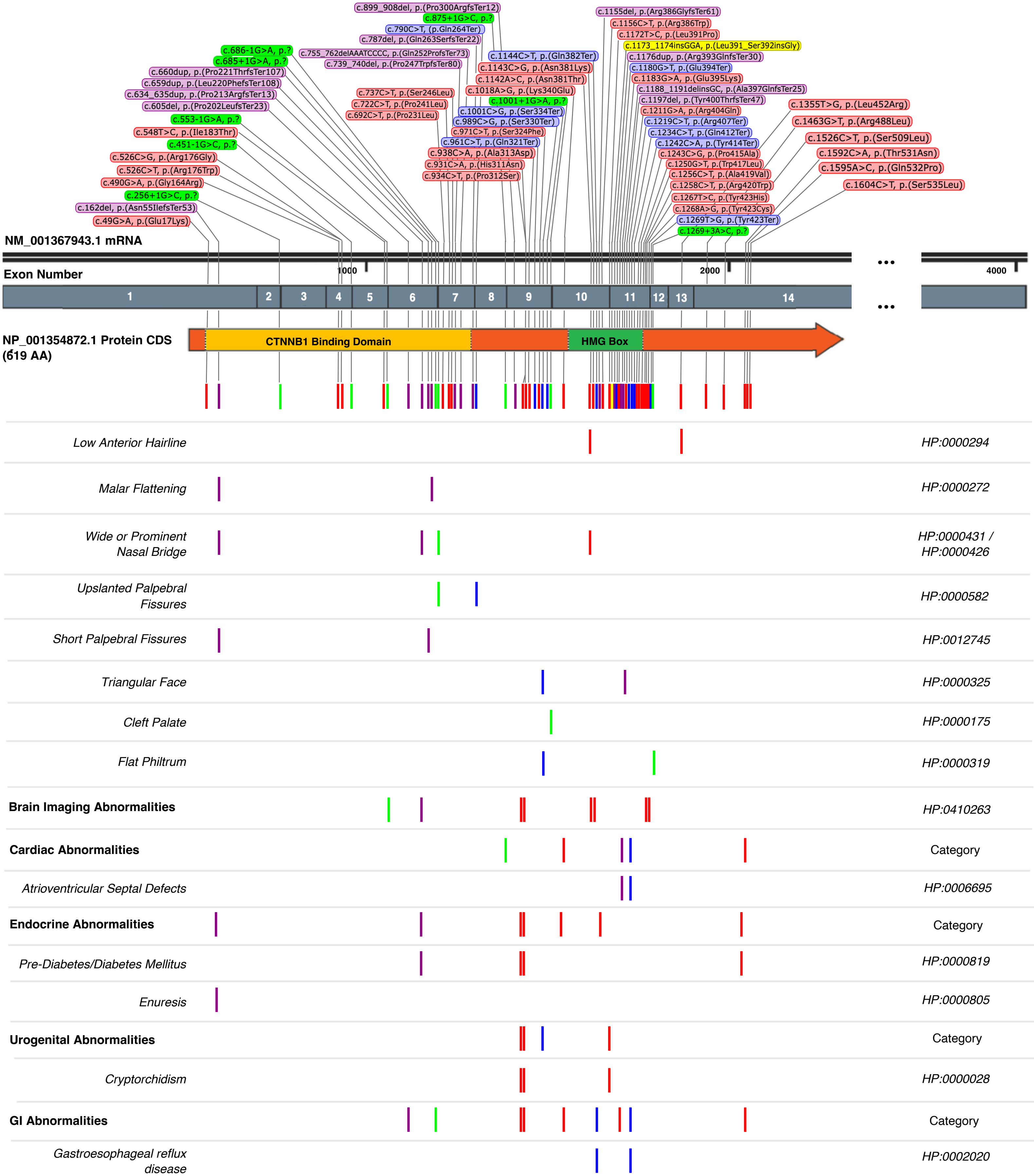

c.899_908del, p.(Pro300ArgfsTer12)
c.875+1G>C, p.?
c.790C>T, (p.Gln264Ter)
c.787del, p.(Gln263SerfsTer22)
c.755_762delAAATCCCC, p.(Gln252ProfsTer73)
c.739_740del, p.(Pro247TrpfsTer80)
c.686-1G>A, p.?
c.685+1G>A, p.?
c.660dup, p.(Pro221ThrfsTer107)
c.659dup, p.(Leu220PhefsTer108)
c.634_635dup, p.(Pro213ArgfsTer13)
c.605del, p.(Pro202LeufsTer23)
c.553-1G>A, p.?
c.548T>C, p.(Ile183Thr)
c.451-1G>C, p.?
c.526C>G, p.(Arg176Gly)
c.526C>T, p.(Arg176Trp)
c.490G>A, p.(Gly164Arg)
c.256+1G>C, p.?
c.162del, p.(Asn55IlefsTer53)
c.49G>A, p.(Glu17Lys)
c.737C>T, p.(Ser246Leu)
c.722C>T, p.(Pro241Leu)
c.692C>T, p.(Pro231Leu)
c.1144C>T, p.(Gln382Ter)
c.1143C>G, p.(Asn381Lys)
c.1142A>C, p.(Asn381Thr)
c.1018A>G, p.(Lys340Glu)
c.1001+1G>A, p.?
c.1001C>G, p.(Ser334Ter)
c.989C>G, p.(Ser330Ter)
c.971C>T, p.(Ser324Phe)
c.961C>T, p.(Gln321Ter)
c.938C>A, p.(Ala313Asp)
c.931C>A, p.(His311Asn)
c.934C>T, p.(Pro312Ser)
c.1155del, p.(Arg386GlyfsTer61)
c.1156C>T, p.(Arg386Trp)
c.1172T>C, p.(Leu391Pro)
c.1173_1174insGGA, p.(Leu391_Ser392insGly)
c.1176dup, p.(Arg393GlnfsTer30)
c.1180G>T, p.(Glu394Ter)
c.1183G>A, p.(Glu395Lys)
c.1188_1191delinsGC, p.(Ala397GlnfsTer25)
c.1197del, p.(Tyr400ThrfsTer47)
c.1211G>A, p.(Arg404Gln)
c.1219C>T, p.(Arg407Ter)
c.1234C>T, p.(Gln412Ter)
c.1242C>A, p.(Tyr414Ter)
c.1243C>G, p.(Pro415Ala)
c.1250G>T, p.(Trp417Leu)
c.1256C>T, p.(Ala419Val)
c.1258C>T, p.(Arg420Trp)
c.1267T>C, p.(Tyr423His)
c.1268A>G, p.(Tyr423Cys)
c.1269T>G, p.(Tyr423Ter)
c.1269+3A>C, p.?
c.1355T>G, p.(Leu452Arg)
c.1463G>T, p.(Arg488Leu)
c.1526C>T, p.(Ser509Leu)
c.1592C>A, p.(Thr531Asn)
c.1595A>C, p.(Gln532Pro)
c.1604C>T, p.(Ser535Leu)
NM_001367943.1 mRNA
1000
2000
4000
...
Exon Number
1
2
3
4
5
6
7
8
9
10
11
12
13
14
NP_001354872.1 Protein CDS
(619 AA)
CTNNB1 Binding Domain
HMG Box
Low Anterior Hairline
HP:0000294
Malar Flattening
HP:0000272
Wide or Prominent Nasal Bridge
HP:0000431 / HP:0000426
Upslanted Palpebral Fissures
HP:0000582
Short Palpebral Fissures
HP:0012745
Triangular Face
HP:0000325
Cleft Palate
HP:0000175
Flat Philtrum
HP:0000319
Brain Imaging Abnormalities
HP:0410263
Cardiac Abnormalities
Category
Atrioventricular Septal Defects
HP:0006695
Endocrine Abnormalities
Category
Pre-Diabetes/Diabetes Mellitus
HP:0000819
Enuresis
HP:0000805
Urogenital Abnormalities
Category
Cryptorchidism
HP:0000028
GI Abnormalities
Category
Gastroesophageal reflux disease
HP:0002020
